# A novel virtual barcode strategy for accurate panel-wide variant calling in circulating tumor DNA

**DOI:** 10.1186/s12859-020-3412-2

**Published:** 2020-04-03

**Authors:** Leilei Wu, Qinfang Deng, Ze Xu, Songwen Zhou, Chao Li, Yi-Xue Li

**Affiliations:** 1 School of Life Sciences and Biotechnology, Shanghai Jiao Tong University, Shanghai, 200240 China; 2 Department of Oncology, Shanghai Pulmonary Hospital, Tongji University School of Medicine, Shanghai, 200433 China; 3 Smartquerier Biomedicine, Shanghai, 201203 China; 4 Shanghai Center for Bioinformation Technology, Shanghai, 201203 China; 5 CAS Key Laboratory of Computational Biology, CAS-MPG Partner Institute for Computational Biology, Shanghai Institute of Nutrition and Health, Shanghai Institutes for Biological Sciences, University of Chinese Academy of Sciences, Chinese Academy of Sciences, Shanghai, 200031 China

**Keywords:** ctDNA, Low-AF SNV, Virtual barcode, Panel-wide calling algorithm, Stochastic noise, Stereotypical noise

## Abstract

**Background:**

Hybrid capture-based next-generation sequencing of DNA has been widely applied in the detection of circulating tumor DNA (ctDNA). Various methods have been proposed for ctDNA detection, but low-allelic-fraction (AF) variants are still a great challenge. In addition, no panel-wide calling algorithm is available, which hiders the full usage of ctDNA based ‘liquid biopsy’. Thus, we developed the VBCALAVD (Virtual Barcode-based Calling Algorithm for Low Allelic Variant Detection) in silico to overcome these limitations.

**Results:**

Based on the understanding of the nature of ctDNA fragmentation, a novel platform-independent virtual barcode strategy was established to eliminate random sequencing errors by clustering sequencing reads into virtual families. Stereotypical mutant-family-level background artifacts were polished by constructing AF distributions. Three additional robust fine-tuning filters were obtained to eliminate stochastic mutant-family-level noises. The performance of our algorithm was validated using cell-free DNA reference standard samples (cfDNA RSDs) and normal healthy cfDNA samples (cfDNA controls). For the RSDs with AFs of 0.1, 0.2, 0.5, 1 and 5%, the mean F1 scores were 0.43 (0.25~0.56), 0.77, 0.92, 0.926 (0.86~1.0) and 0.89 (0.75~1.0), respectively, which indicates that the proposed approach significantly outperforms the published algorithms. Among controls, no false positives were detected. Meanwhile, characteristics of mutant-family-level noise and quantitative determinants of divergence between mutant-family-level noises from controls and RSDs were clearly depicted.

**Conclusions:**

Due to its good performance in the detection of low-AF variants, our algorithm will greatly facilitate the noninvasive panel-wide detection of ctDNA in research and clinical settings. The whole pipeline is available at https://github.com/zhaodalv/VBCALAVD.

## Background

Somatic mutations play key roles in human diseases, such as cancer [1] and neurological disease [2]. In cancer, these mutations can be raw materials for cancer evolution [3, 4] and serve as actionable targets [5–7]. Thus, many variant calling algorithms, such as Varscan2, MuTect, and SiNVICT, have been developed for accurate detection of somatic mutations through next-generation sequencing [8–12]. Several studies have benchmarked the performance of various somatic variant callers [13–17], and most of these studies assess performance at an allelic fraction (AF) level greater than 1%. However, systematic benchmarking and assessment studies using high-sequencing-depth samples with an AF as low as 0.1% have not been performed. A previous study showed that notably different sensitivities were found even at the 5% AF level, and the callers achieved low positive predictive values (PPVs). At a high AF level (5% ~ 100%), although the PPVs increase as AF rises, the PPVs continue to show substantial fluctuations among the tested calling algorithms [14]. Another study demonstrates that the published approaches yield unreliable results for an AF as low as 2% [16]. Thus, the detection limits of most calling algorithms restrict their calling efficacy of low-AF variants, which might be problematic in several applications, particularly liquid biopsies due to the low template levels of mutant circulating tumor DNA (ctDNA) in patient plasma [18, 19].

The detection limit of single nucleotide variants (SNVs) is generally affected by the input DNA quantities and sequencing depth [20, 21]. A high sequencing depth is required for the detection of low-AF variants. However, the coverage increment accompanies with an increase in the background error rate. Many methods have been proposed to suppress this type of background noise. These methods include the use of exogenous molecular barcodes (unique molecular identifiers, UMIs), endogenous position-based method, sequencing technical replicates [20, 22–26] and background error modeling [20, 26, 27]. The UMI strategy is an effective way to remove stochastic sequencing errors [20, 28–30] and duplicates, which can improve the accuracy of low-frequency variant detection and solve severe quantitative bias in RNA-seq [31]. However, UMIs’ universal application is limited by their experimental design [32]. The endogenous position-based method is an alternative way to deal with duplicates and remove errors. Modules in popular tools such as SAMtools [33] and Picard (http://broadinstitute.github.io/picard/) use this approach to mark duplicates, select a representative read and further improve calling results and RNA quantification [31]. However, these tools are based on 5′ prime position of a read and do not use full segment information. In addition, usage of a representative read with the highest mapping quality or total base quality could result in a false call at a specific genomic position. Application of sequencing technical replicates in ERASE-Seq can significantly eliminate stochastic errors [27]. However, its application involves greater expense than the single-replicate method, and some low-AF stochastic errors cannot be removed without replicates.

Background error modeling as a complementary method can eliminate recurrent/stereotypical errors well. The proposed methods apply different kinds of samples to construct background models, such as tumor samples in TAm-Seq [26], healthy cfDNA samples in iDES [20] and sample replicates in ERASE-Seq [27]. ERASE is limited by sample replicate numbers, and the likelihood that stereotypical noise position changes over sequencing time is certain. For TAm-Seq, background models from tumor samples limit its usage in ultradeep cfDNA data. iDES does not consider the impact of different cfDNA templates on stereotypical errors, as the cfDNA quantities of patients with various types of cancer are far higher than those of normal healthy individuals [34–37].

For various applications, many UMI-based calling strategies and computer pipelines have been developed [20, 24, 30], but these studies are primarily limited to the detection of mutations in COSMIC and hotspot sites. Only two different panel-wide calling methods have been proposed for ctDNA detection: iDES and SiNVICT [10, 20]. iDES provides pipelines for input file conversion, background database construction, background polishing, and quality control statistics but does not incorporate a detailed panel-wide calling method. The performance of SiNVICT is validated based on simulated and real data, but the analysis of its performance based on real data only focuses on the sensitivity aspect. The panel-wide calling ability of SiNVICT requires validation using a large panel. Accurate panel-wide mutation calling is essential for various clinical applications, such as calculation of the tumor mutation burden (TMB) [38, 39], which serves as an important marker for immunotherapy and a significant indicator for gene fusion detections [40]. Thus, an effective panel-wide calling method needs to be established.

**Fig. 1:**
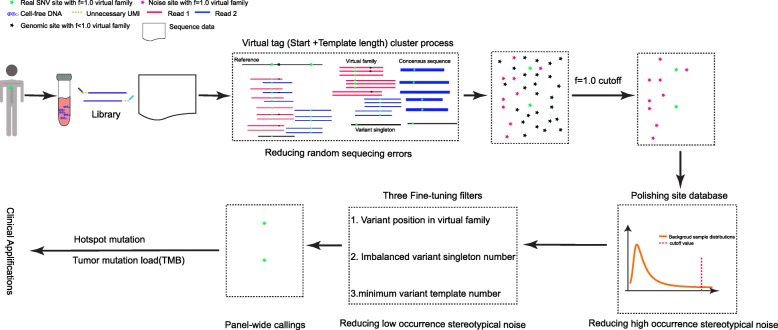
Flowchart of the virtual barcode based calling algorithm for low allelic variant detection

To address these problems, we performed a comprehensive analysis of the sources of background noise generated by cfDNA sequencing data from normal health plasma (controls) and reference standard samples (RSDs). Based on that, a novel virtual barcode was first established on high-depth cfDNA data. Unlike the previous position-based deduplication method, our virtual barcode was based on segment position and length, which would help us to distinguish different segments with the same 5′ position but different 3′ positions. Like UMI, virtual barcode was used to cluster reads to form a consensus sequence, which provides a more reliable base call at every position. Unlike previously proposed endogenous unique identifiers [23], the performance of virtual barcode was comprehensively validated in exogenous UMI cfDNA samples, which made our algorithm platform-independent and universally applicable for the noninvasive detection of SNVs using next generation sequencing (NGS) data in silico. As illustrated in Fig. 1, our algorithm first utilizes a virtual barcode to eliminate sequencing errors, polishes the stereotypical background artifacts among background samples (BGs) and then uses three fine-tuning filters to achieve great sensitivity and specificity. We anticipate that the proposed algorithm will have wide applications in research and clinical settings.

## Results

### Performance of our virtual barcode

**Fig. 2:**
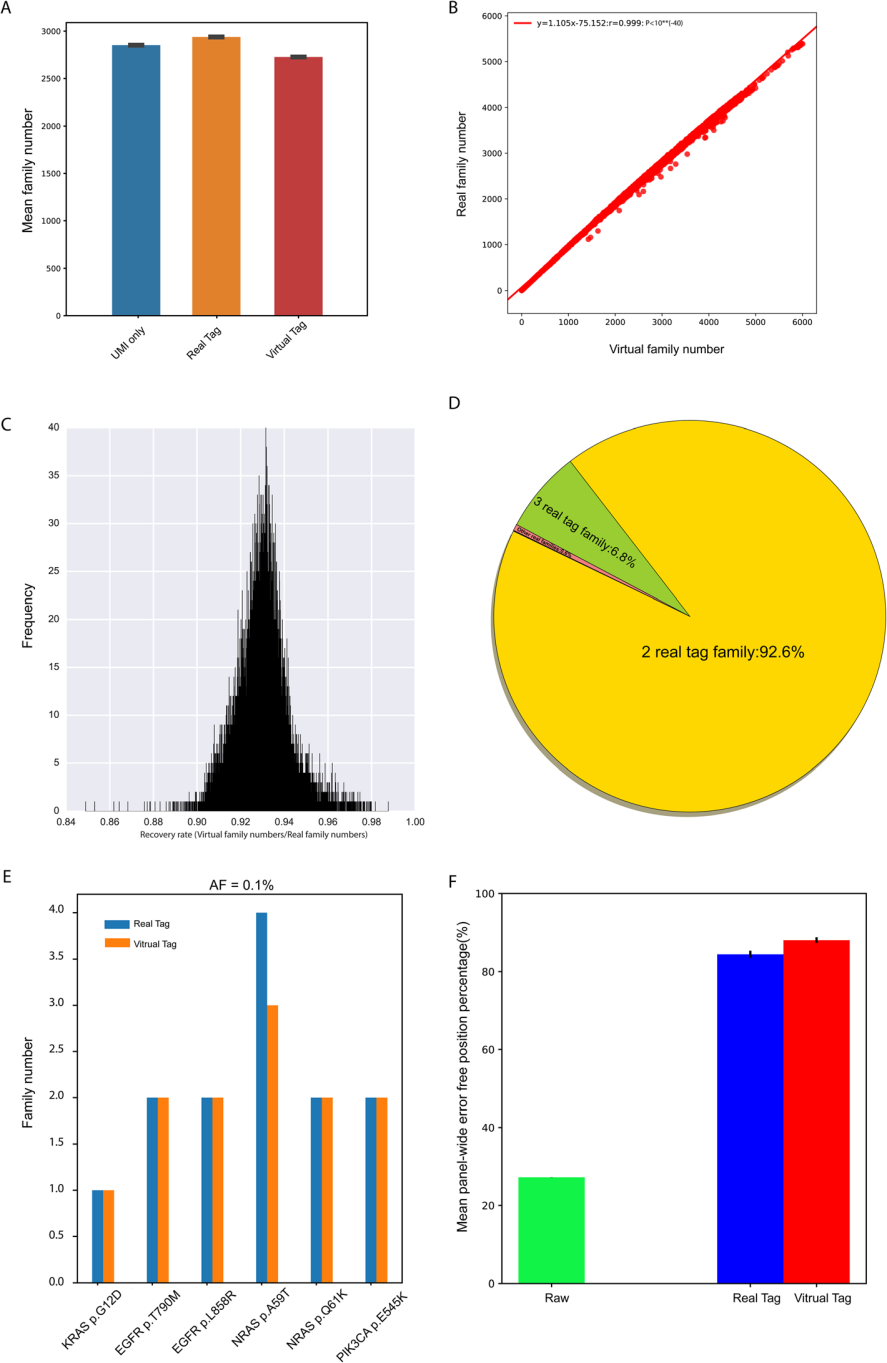
Comparison between the virtual tag and real tag using 3 Oncosmart2 UMI samples. **a** Mean family numbers and corresponding SD from 10 samples (20,000 random genomic positions per sample) obtained using the UMI alone (blue bar), the real tag (orange bar) and the virtual tag (red bar). **b** Significant linear relationship between virtual family numbers and real family numbers for 20,000 genomic positions (R^2^ = 1.0). **c** Recovery rate distribution for real family numbers at 20,000 genomic positions. **d** Percentage of wrongly clustered virtual family calculated by different assigned numbers of real family. **e** Comparison between f = 1.0 virtual family numbers (orange bar) and f = 1.0 real family numbers (blue bar) among six positive sites in one UMI sample with an AF level of 0.1%. **f** Mean fraction and corresponding SD of the panel-wide error-free position before (green bar) and after application of the real tag (blue bar) and the virtual tag (red bar) in 3 UMI samples

The real family was clustered by UMI, start site and template length. The virtual family was defined as reads that shared the same start site, template length and strand. The mean virtual family numbers were slightly fewer than the mean real family numbers (2730 vs. 2943) (Fig. 2a, red bar vs. yellow bar) among 10 samples, and a strong linear relationship was found between virtual and real family numbers among 20,000 randomly selected genomic positions in one sample (Fig. 2b; y = 1.105x - 75.152, 95% Confidence interval (CI): 1.1038~1.1058, *P* < 10^− 40^; R^2^ = 100%). The recovery rates for real families among the majority of the 20,000 positions ranged from 91.87 to 94.0% (Fig. 2c; 92.98% ± 1.1%) and only a small proportion of reads with different UMI tags were mistakenly clustered by the virtual barcode. The incorrectly clustered family contents were investigated. The results showed that 92.6% of these members were composed of two real families, and 6.8% were three real families (Fig. 2d). The incorrect clusters might introduce false negatives, particularly if the allele number of a variant is extremely low. Thus, we compared f = 1.0 virtual family numbers with f = 1.0 real family numbers at six positive sites among three UMI samples. At the 0.1% level, five out of the six positive sites had equal family numbers and no false negatives were detected (Fig. 2e). Similar to the 1 and 5% levels, no false negatives were found (Figure S1). At the same time, AF values of six positive sites calculated from the virtual-family level were close to the expected AF values and similar to the AF values from the variant-read level (Figure S1). In the decreasing noise aspect, efficiencies of the virtual tag and real tag were the same, supported by similar mean fraction of panel-wide error-free genomic positions (Fig. 2f; Real tag: 84.44% ± 0.91%; Virtual tag: 88.07% ± 0.66%) and mean panel-wide error rates (Real tag: 7.1*10^− 5^ ± 0.3*10^− 5^; Virtual Tag: 5.9*10^− 5^ ± 0.5*10^− 5^).

In conclusion, our virtual barcode was sufficiently robust to replace a real UMI tag and could become a universally applicable approach for reducing noise in cfDNA sequencing samples.

**Fig. 3:**
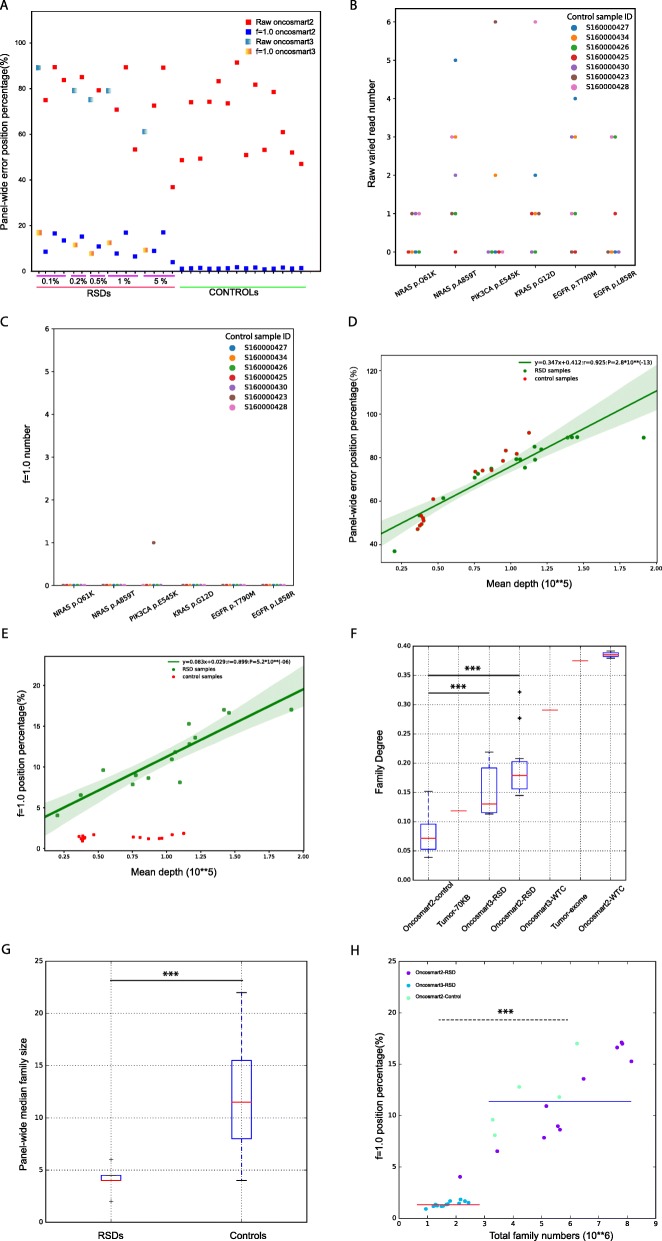
Noise profile among the 30 background samples (BGs) before and after application of the virtual barcode. **a** Panel-wide error position percentage in every BG before and after application of the virtual barcode (Oncosmart2 BGs: blue square to red square; Oncosmart3 BGs: gradient blue to gradient yellow). **b** Numbers of non-reference alleles at six positive sites among the top 7 high-sequence-depth controls at six positive sites. **c** Numbers of the variant f = 1.0 virtual family at six positive sites among the top 7 high-sequence-depth controls. **d** Significant linear relationship between the panel-wide mean depth and the panel-wide error position percentage among 30 BGs (green dot: 16 RSDs; red dot: 14 controls; R^2^ = 85.56%). **e** Relationship between the fraction of the error position with f = 1.0 virtual family and the panel-wide mean depth among 30 BGs after application of the virtual barcode (16 RSDs, green dots; 14 controls, red dots). A significant linear relationship was observed in the 16 RSDs (R^2^ = 80.82%). **f** Boxplot of family degree for 11 Oncosmart2 RSDs, 5 Oncosmart3 RSDs, 14 Oncosmart2 controls, HWT samples and 2 tumor samples. Compared with controls, the Oncosmart2 and Oncosmart3 RSDs had significantly higher family degrees; *** means *P* < 0.001. **g** Boxplot of panel-wide median family size between 14 controls and 16 RSDs; *** means *P* < 0.001. (H) Significantly higher error percentage in the 16 high-template RSDs (blue line) than in 14 low-template controls (red line)

Subsequently, virtual barcode was applied for 30 BGs, and the panel-wide error position percentage was significantly decreased in every BG (Fig. 3a). In turn, the mean panel-wide error-free position percentage was improved by ~ 64.11% ± 12.9%. The ability of the method to decrease random read errors was further confirmed at six positive sites in the top 7 high-sequencing-depth control samples. There were random non-reference alleles in two or more samples at the positive site (Fig. 3b), and nearly all of these alleles were eliminated (Fig. 3c). These results confirmed the good and stable performance of our virtual barcode for decreasing read-level stochastic noise.

### Characteristics of mutant-family-level noise

A small proportion of error sites supported with f = 1.0 mutant families made the virtual barcode/real tag alone indistinguishable from real variants. We denote this type of noise mutant-family-level noise (designated as f = 1.0 sites). Thus, additional robust filters are needed to improve the specificity of the proposed algorithm.

The profiles of mutant-family-level noise among 14 controls and 16 RSDs showed an interesting divergence. A significant linear relationship between the mean depth and error position percentage (Fig. 3d; y = 0.347x + 0.412, 95% CI: 0.292~0.402, *P* = 2.8*10^− 13^; R^2^ = 85.56%) remained at the mutant-family-level in the RSDs (Fig. 3e, green line; y = 0.083x + 0.029, 95% CI: 0.059~0.107, *P* = 5.22*10^− 6^; R^2^ = 80.82%;) but not among the controls (Fig. 3e, red dots). This disagreement might be caused by input DNA quantities (virtual family numbers) and uneven depth/coverage. By normalizing panel-wide virtual family numbers based on coverage, the family degree was obtained for every sample. Compared with controls, the median virtual family degree was significantly higher in both Oncosmart2 (2.49-fold, *P* = 2.26*10^− 5^) and Oncosmart3 RSDs (1.88-fold, *P* = 0.007; Fig. 3f). Based on the observation that the reciprocal of family degree could reflect panel-wide median virtual family size (Figure S2), 14 controls had significantly larger overall virtual family size than 16 RSDs (Fig. 3g; *P* = 5.88*10^− 5^), which in turn could give more confident support for calculating f values and further decreasing random read-level noise (Fig. 3f; Figure S2). The significantly larger family size in 14 controls was caused by the significantly lower template numbers than 16 RSDs (*P* = 2.05*10^− 5^, Figure S2). The scatterplot clearly showed that high template numbers in 16 RSDs caused a significantly higher percentage of mutant-family-level noise than 14 controls (*P* = 6.25*10^− 8^; Fig. 3h). This result indicated that using cfDNA data from normal healthy individuals with low-level templates as the background [20, 41] is not sufficient to cover all noises in samples with high-level templates under similar sequencing coverage. Thus, we combined controls with RSDs for the following analysis.

**Fig. 4:**
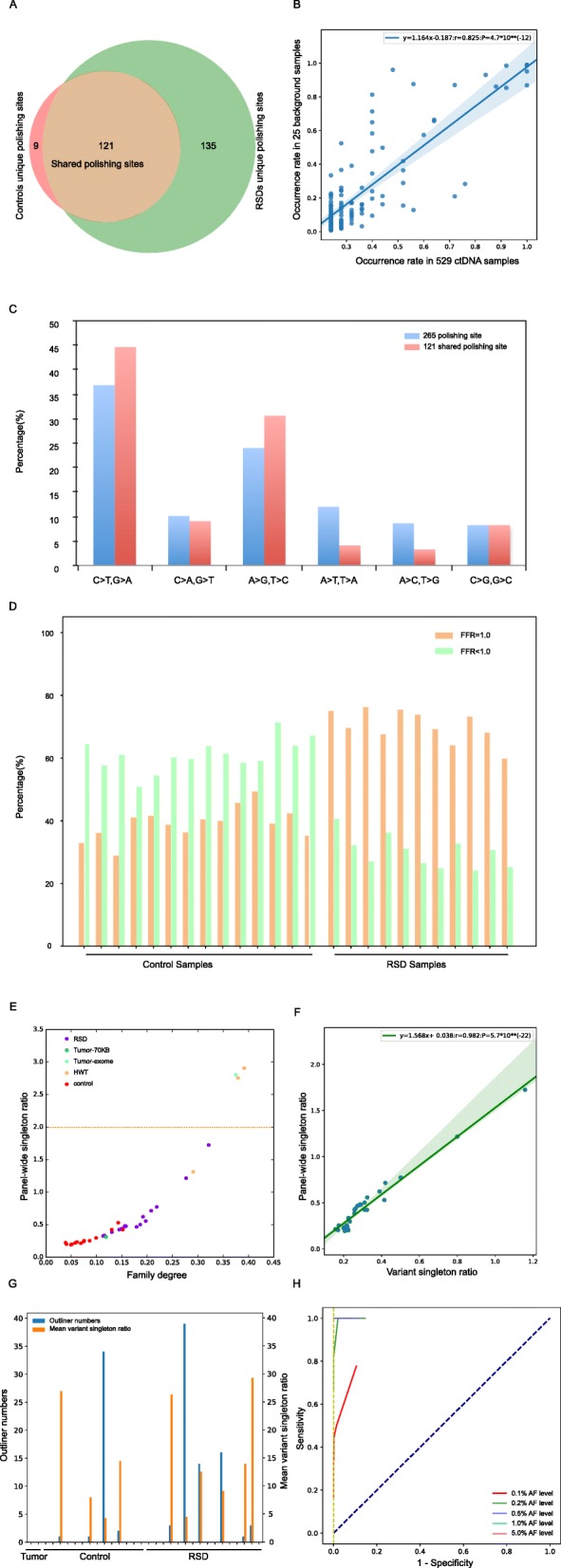
Stereotypical noise characteristics and effectiveness of fine-tuning filters. **a** Stereotypical site numbers from 14 Oncosmart2 controls and 11 Oncosmart2 RSDs: 121 shared sites among the 14 Oncosmart2 controls and 11 Oncosmart2 RSDs (brown region), nine sites from only from the controls (red region), and 135 sites only from the RSDs (green region). **b** Significant linear relationship between the incidence rate in 25 Oncosmart2 BGs and the incidence rate in 529 Oncosmart2 cfDNA samples among 121 shared polishing sites (R^2^ = 67.8%). **c** Percentages of 12 substitution types among 265 polishing sites (blue bar) and 121 shared sites (red bar). **d** Fraction of positions that completely consisted of false families (orange bar) at stochastic f = 1.0 site in every Oncosmart2 BG sample. **e** Direct correlation between family degree and panel-wide singleton ratio among all samples (dashed line represents 2.0). **f** Significant linear relationship (R^2^ = 96%) between panel-wide singleton ratio and mean variant singleton ratio from high-AF sites (AF > =0.05) among 30 BGs. **g** Effectiveness of sample-level strategy to remove variant singleton ratio outliners at the FDR < 0.01 level for all samples; Blue bar: filtered numbers; Orange bar: the corresponding mean variant singleton ratio. **h** ROC curve based on the optimal template feature (updated f = 1.0 virtual family numbers plus qualified variant singletons) at every AF level under a theoretical confidence level ranging from 80 to 99.5%

According to the relationship between sample occurrence and AF spectra (Figure S3), mutant-family-level noises were classified into two types: stereotypical (occurrence > = 6 BGs) and stochastic mutant-family-level noise. In total, we obtained 265 unique stereotypical variants (Fig. 4a). The RSDs made a greater contribution than the controls to recovering stereotypical variants, many of which occurred only once in controls (Figure S4). As expected, 265 stereotypical noises occurred stably showing a significant linear relationship between 25 Oncosmart2 BGs and 529 Oncosmart2 cfDNA samples (Figure S3; y = 1.097x - 0.137, 95% CI: 0.922~1.235, *P* = 5.6*10^− 32^; R^2^ = 41.7%). Further analysis of the occurrence rates of 121 shared noises (Fig. 4a) showed a significant linear relationship with a higher R^2^ value (Fig. 4b; y = 1.164 x – 0.187, 95% CI: 1.019 ~ 1.308, *P* = 4.7*10^− 12^; R^2^ = 67.8%). Additionally, after polishing based on Oncosmart2, no stereotypical noises were found among the 5 Oncosmart3 RSDs at the intersection region of the two panels (Table S2–2). Stereotypical noise is caused by many factors, such as DNA damage [42] and PCR errors [43], which have different substitution preferences. The main substitution types of our stereotypical variants were C > T/G > A, C > A/G > T, and A > G/T > C (71.05%, Fig. 4c), which were consistent with the substitution types from Oncosmart3 RSDs (Table S2–4) and previously reported error profiles for ‘Kapa HF’ polymerase [43]. The percentage of these six substitutions further increased to 84.297% in 121 shared sites, which demonstrated that these substitutions introduced by PCR errors were likely to occur universally (Fig. 4b, Figure S3; R^2^:67.8% vs. 41.7%). These PCR-induced distortions are mainly caused by PCR stochasticity and polymerase errors [43, 44] and cannot be removed by UMI strategies only [20, 43].

### Strategies for decreasing mutant-family-level noises

Based on a clear understanding of the characteristics of stereotypical noise, a filtered database was constructed for the polishing of real mutations of the same type at these sites (265 polishing sites). Unlike in the previously proposed iDES polishing method [20], we first obtained 10 best-fit candidate distributions from 529 Oncosmart2 cfDNA samples based on AIC, BIC, SEE, and R values, which were independently validated in 104 Oncosmart1 cfDNA samples. Then a comparison between the iDES construction step and our step was made (Figure S4). Finally, 265 stereotypical variants were polished by calculating cutoff AF values from the best-fitted personalized distribution. The results showed that the ‘Johnsonsu’ distribution was the best-fitted distribution (Table 1; 26%). AF cutoffs are shown in Table S3–3.

**Table 1:** Information on the best distribution among 265 polishing sites

Distributions	Best numbers	Percentage (%)	Mean sample size	Sample size range
Dweibull	11	4.15	21.181818	8~95
Lognorm	18	6.79	108.052632	19~354
Alpha	19	7.17	104.157895	8~475
Exponnorm	24	9.06	122.791667	21~545
Weibull_min	25	9.43	89.16	7~550
Nct	27	10.19	173.962963	9~514
Gamma	33	12.45	139.757576	8~525
Beta	39	14.72	139.794872	6~479
Johnsonsu	69	26.04	109.115942	8~529

Compared with stereotypical noises, stochastic mutant-family-level noises (designated as stochastic f = 1.0 site) were prone to low AF values, wide AF value spectra and unstable occurrence (Figure S3). Three additional fine-tuning filters were proposed based on appropriate specific features.

The minimum absolute distance (Ds value) was obtained between the distances from the variant position to the start and end positions in the corresponding virtual family. Ds trajectories of f = 1.0 families from the stochastic f = 1.0 site were compared with Ds trajectories from high-AF sites, positive sites, mutant singletons, and Ds trajectories of f < 1.0 virtual families from genomic sites filtered by the virtual barcode step (Figure S5). Then, the specific Ds value (<=2 and > =149) for stochastic f = 1.0 site was obtained. The virtual family that met the identified Ds value was defined as a false family. In every BG, the percentage of sites fully constituted by a false family (false family ratio: FFR = 1.0) was calculated and is shown as an orange bar in Fig. 4d and Figure S9.

With respect to the variant singleton ratio, based on the observation that variant singleton numbers (ranging from 0 to 39) among stochastic f = 1.0 sites were significantly higher than variant singleton numbers among six positive sites, we hypothesized that for the real SNV site, the ratio of singleton numbers to f = 1.0 family numbers would fluctuate within a certain range. First, at the panel level, the singleton ratios of all BGs were less than 2.0 (Fig. 4e). This singleton ratio was a general robust cutoff value that could well distinguish positive mutations, known mutations of non-small-cell lung carcinoma (NSCLC) patients [45] and high AF variants from these stochastic family-level noises (Figure S6). Second, at the sample level, the mean variant singleton ratios of high-AF sites could reflect the panel-wide singleton ratio, indicating that the variant singleton ratios of real variants fluctuated around the panel-wide singleton ratio (Fig. 4f). Thus, a sample-level strategy based on the distribution of singleton ratios from high-AF variants (AF > =0.05) was applied (Figure S6). After false discovery rate (FDR) correction, a small number (blue bar) of extreme outliners with mean ratios ranging from 4.1~28.2 (orange bar) were removed (FDR < =0.01; Fig. 4g). In addition, our method was relatively conservative, and no outliers were found in samples with an overall high or low singleton ratio (Figure S6), such as two tumor samples (Fig. 4g). In conclusion, this filter could avoid over-recovery of variant singletons at genomic sites vulnerable to random noise.

Finally, template numbers were updated and updated f = 1.0 numbers and qualified variant singletons were obtained. This updated template feature was the most specific features (Figure S7). Based on this specific template feature, an ROC curve was constructed for six positive sites at every AF level (Fig. 4h), which showed an optimal tradeoff between sensitivity and specificity at a strict 99% confidence level.

### Effectiveness of all the filters in improving the panel-wide calling efficacy

**Fig. 5:**
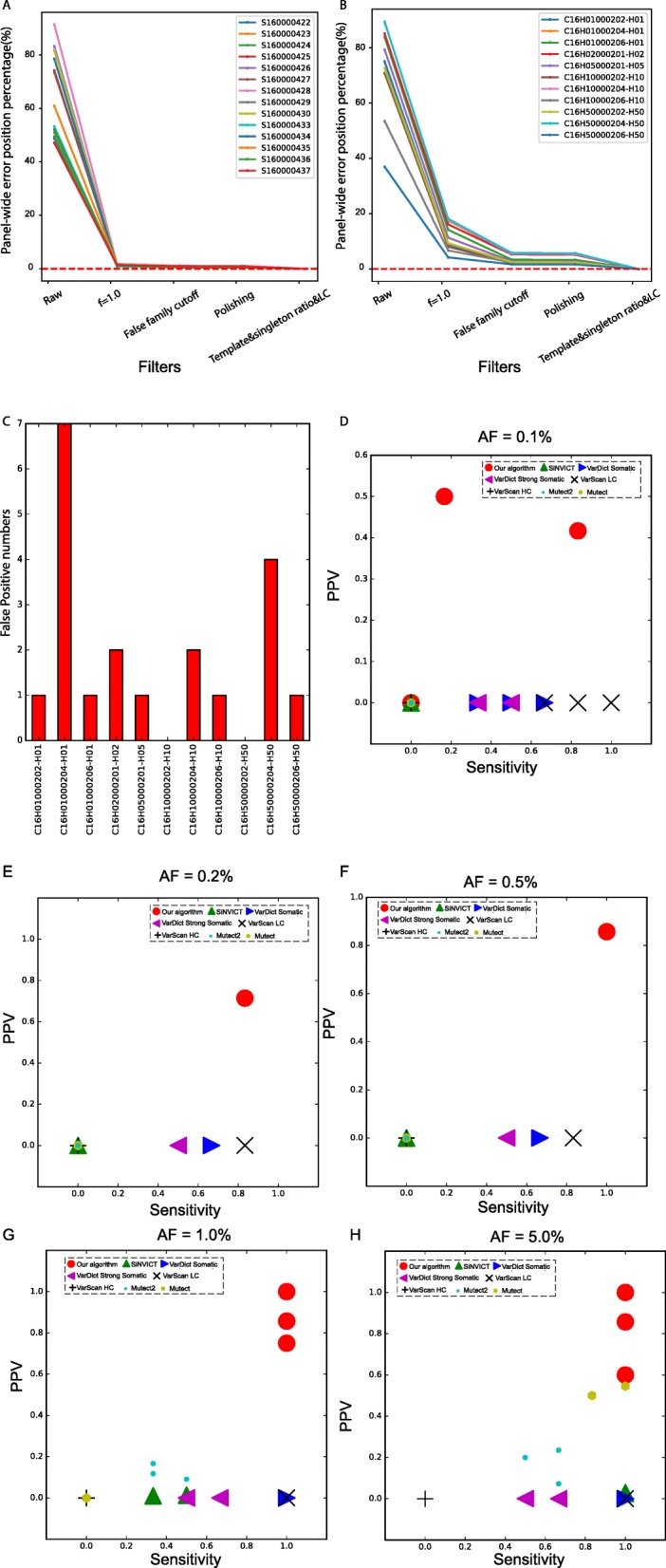
Systematic evaluation of the effectiveness of all filters. **a**, **b** Fraction of panel-wide error-free positions in the 14 Oncosmart2 controls and 11 Oncosmart2 RSDs obtained with each filter. **c** Numbers of false-positive sites retained among 11 Oncosmart2 RSDs. **d**–**h** Panel-wide sensitivity and PPVs obtained with our algorithm (red circles) and five published calling algorithms using Oncosmart2 RSDs with AF values ranging from 0.1 to 5%

We systematically evaluated the effectiveness of each of the above-described three steps in the proposed approach. With respect to reducing noise, the virtual barcode clustering step removed the majority of noise in both 14 Oncsmart2 controls (Fig. 5a) and 11 Oncsmart2 RSDs (Fig. 5b). The subsequent filters showed greater effectiveness of error reduction in RSDs versus controls (Fig. 5b), indicating the necessity of these filters for error reduction in high-template samples, such as samples from various types of cancer. By combining all the filters, the mean panel-wide error position percentage of 25 Oncosmart2 BGs was extremely low (Table S4; 7.95*10^− 4^%), lower than reported percentage in iDES (2%~ 10%). In Oncosmart2 RSDs, false-positive sites were maintained at extremely low numbers (Fig. 5c). We then calculated the sensitivity, PPV, F1 score and false positive rate (FPR) per genomic position of our algorithm and five panel-wide calling algorithms at every level using 25 Oncosmart2 BGs (Figure S8; Table S4). The results showed that the performance of our algorithm was significantly better than that of previously published calling software at every AF level from 0.1 to 5% (Figs. 5d-h). Our algorithm kept the false positive rate (FPR) per genomic position lower than benchmarked software and the reported FPRs in ERASE-seq [27] and iDES (Table S4). Additional validation of our algorithm using 5 Oncosmart3 RSDs proved the robustness of our algorithm at AF levels ranging from 0.1 to 5% (Figure S9; Table S2–1: Sensitivity).

A small number of false-positive sites were retained in the 25 Oncosmart2 BGs. From a previous reference, we incorporated low-complexity (LC) regions [46] and short tandem regions (STRs) [47] into the pipeline. False-positive sites left in controls were annotated as SNP sites (Table S5) and explained by the “spreading-of-signal” [48] with the newer sequencing platform (HiSeq 3000/4000/X Ten) in the same sequencing lane (Table S6).

## Discussion

Recently, several studies have focused on the application of cfDNA fragmentation information in clinical settings [49–51]. Here, for the first time, we use cfDNA fragmentation information as an endogenous UMI to decrease random sequencing noise. A previous study showed that a similar endogenous UID (unique identifier) can be applied to decrease random sequencing noise, though it relies heavily on random DNA or RNA fragmentation [25, 32]. Through comprehensive validation from exogenous UMI cfDNA data and supported by application in our previous research [45], our endogenous UMI fit cfDNA well.

The downside of this step was that approximately 8% of the UMI was wrongly clustered by the virtual barcode, because different cfDNA molecules have a certain probability of sharing the same virtual barcode [19]. This downside of our proposed method leads to a lower yield of usable families that might generate lower f = 1.0 supported family numbers for a candidate mutation, as shown by the lower f = 1.0 virtual family numbers compared with f = 1.0 real family numbers in Fig. 2e and Figure S1. This downside did not have an effect on the sensitivity or PPV at any of the AF levels tested in this study, and thus, we did not further optimize this step of the algorithm. However, because this downside might have some effect in some cases, the value of the f parameter can be adjusted to minimize this effect. This step can also be affected by paralogous sequences. Reads in these regions tend to have lower mapping quality due to multiple alignments. Multiple mismatches (MM) [52] are another feature to avoid this effect.

For the polishing step, unlike iDES, we found the most best-fitted distribution of stereotypical noise through large samples. Meanwhile, best-fitted distributions also provided informative prior distributions for distribution construction with low sample sizes using Bayesian methods.

For the variant singleton ratio filter, the hypothesis of this filter relies on the panel-wide singleton ratio and sequencing depth (family degree). For samples with panel-wide singleton ratios lager than 2, this calculation process might not be necessary. For example, for one exome dataset, most of its templates were singletons (Figure S6) that were the main virtual family form to support variants. Under this circumstance, overall variant singleton ratios were high among the variants. Besides the panel-wide singleton ratio, sequence depth is another factor. For the tumor-70 kb panel with extremely low sequence depth among all samples (Figure S2), its low family degree under low sequence depth led to a small proportion of singletons that caused overall low variant singleton ratios (Fig. 4e: dark green dot; Figure S6). Although our method can intelligently recognize these samples, we though that there should be a sample level cutoff value to assess whether this sample needs the calculation process of this filter, and related precise sample level cutoff values need further detailed investigation in large series of family degree samples with different sequencing depths.

## Conclusions

This study develops a novel calling algorithm for the accurate detection of somatic mutations with an AF as low as 0.1%. The algorithm introduces three noise-reduction strategies based on a comprehensive analysis of the source of different types of sequencing noise. The robustness of the strategies is well elaborated using 11 Oncosmart2 RSDs and 14 Oncosmart2 controls and validated with 5 Oncosmart3 RSDs. Our algorithm is independent of the platform and well suited for NGS data with or without a UMI. Due to its good performance for the detection of low-AF mutations, our algorithm will greatly facilitate the noninvasive panel-wide detection of ctDNA in research and clinical settings.

## Methods

### Materials

In the present study, the following materials were included: 14 Oncosmart2 cfDNA samples (controls) from healthy individuals, 16 cfDNA reference standards (RSDs, HD780), 529 Oncosmart2 patient cfDNA samples, 104 Oncosmart1 patient cfDNA samples, 2 tumor samples, and 3 wild-type cfDNA samples (HWT). RSDs were harboring six SNV-positive sites with AF levels 0.1% (4 samples), 0.2% (2 samples), 0.5% (2 samples), 1% (4 samples) and 5% (4 samples). Three of RSDs were UMI samples with AF 0.1, 1 and 5%. Our background samples (BGs) were 14 controls and 16 RSDs. We further classified BGs with respect to their panel version. Fourteen controls and 11 Oncosamrt2 RSDs made up the 25 Oncosmart2 BGs and were used to set and optimize the filters used in our algorithm. Five Oncosmart3 RSDs (per sample at every AF level) were Oncosmart3 BGs that we used to validate all the filters constructed based on the analysis of the 25 Oncosamrt2 BGs. Three Oncosmart2 UMI RSDs were used to validate the effectiveness of our virtual barcode. For 2 tumor samples, one was enriched in the 70 kb panel, and one was whole-exome data. These 2 tumor samples were used as internal standards for family degree exploration. Sample statistics after preprocessing are provided in Table S1.

A total of 529 Oncosmart2 patient cfDNA samples and 104 Oncosmart1 patient cfDNA samples were analyzed for two purposes. First, the stability of the occurrence rate for selected stereotypical sites was validated using 529 Oncosmart2 cfDNA samples. Second, we explored the best-fit distribution candidates through random position selection using 529 Oncosmart2 cfDNA samples, which were independently validated using 104 Oncosmart1 cfDNA samples. Based on distribution candidates, the distribution for every stereotypical site was built using AF values from 25 BGs and 529 Oncosmart2 samples. All RSDs were used to benchmark five published calling algorithms, and 11 Oncosmart2 RSDs were used to compare the performance of our algorithm with that of five published calling algorithms. More detailed sample descriptions are provided in the Supplementary Methods.

### Virtual barcode-based algorithm

The sequencing reads were clustered into virtual families according to the start site, template length and strand. We validated the robustness and effectiveness of the virtual barcode using 3 Oncosmart2 UMI samples from three aspects: 1) recovery rate of the real family from the UMI; 2) family contents; and 3) effectiveness in suppressing errors. For validation, we randomly selected genomic positions on Oncosmart2 panel 10 times (20,000 positions per sample). After validation, if both read1 (R1) and read2 (R2) from the sample template covered a genomic site, we further consolidated the R1 and R2 families. For a particular genomic site, if the bases from R1 and R2 were the same, only one read was retained in the corresponding virtual family; otherwise, both reads were discarded. The virtual barcode was then defined based on the start site and template length. Consensus reads were reads sharing the same virtual barcode, and at least 2 reads were required for the virtual family. We calculated f value, which is the ratio of the non-reference allele for every family. For a singleton, only the variant singleton was retained if the position had at least one virtual family with f = 1.0.

### Construction of the polishing distribution

To establish a well-fitted distribution for stereotypical mutant-family-level noises (designated as stereotypical f = 1.0 site), we adopted a novel strategy consisting of two steps: 1) identifying candidate distributions from 529 Oncosmart2 cfDNA samples and validating the candidates in 104 Oncosmart1 cfDNA samples independently; and 2) constructing the best-fit distribution for a specific polishing site.

### Additional fine-tuning filters

Based on comprehensive knowledge of the sources of stochastic mutant-family-level noises, three fine-tuning filters were introduced: 1) variant position in a segment, 2) imbalanced singleton number, and 3) minimum template number requirement.

Detailed methods and illustrations of every part are provided in the online Supplementary Methods.

## Supplementary information

**Additional file 1.** **Figure S1.** Non-reference template numbers and AF values calculated from different template features for six positive sites in RSDs.

**Additional file 2.** **Figure S2.** Relationships among virtual family degree, singleton ratio and median virtual family size, covered reads and sequencing depth among thirty BGs, three HWTs, and two tumor samples panel-widely.

**Additional file 3.** **Figure S3.** Characteristics of mutant-family-level noises.

**Additional file 4.** **Figure S4.** Sources of polishing sites and comparisons with iDES.

**Additional file 5.** **Figure S5.** Distance distributions (Ds) and false family ratio (FFR) distributions at four kinds of site and variant singletons.

**Additional file 6.** **Figure S6.** Imbalanced variant singleton ratios.

**Additional file 7.** **Figure S7.** Theoretical detection limit and ROC curve based on different template features.

**Additional file 8.** **Figure S8.** Sensitivity and PPV in relation to AF values from 0.1% to 5% in case of Mutect (yellow circle), Mutect2 (green circle), SiNVICT (green triangle), VarScan2 (Low confidence: black cross; High confidence: black plus), VarDict (blue triangle: somatic; purple triangle: strong somatic).

**Additional file 9.** **Figure S9.** Validation of every filter using 5 Oncosmart3 RSDs.

**Additional file 10.** **Table S1.** (Table S1): Panel-wide statistics for 30 BGs, 3 HWTs and 2 tumor samples.

**Additional file 11.** **Table S2.** (Table S2).

**Additional file 12.** **Table S3.** (Table S3).

**Additional file 13.** **Table S4.** (Table S4).

**Additional file 14.** **Table S5.** (Table S5): Annotations for left sites in controls.

**Additional file 15.** **Table S6.** (Table S6): Distribution of controls’ left sites with AF values in multiplexing samples at same sequencing line.

**Additional file 16.** Supplementary methods.

## Data Availability

All data generated or analyzed during this study are included in this published article and its supplementary information files.

## Abbreviations

95% CI: 95% confidence interval
AF: Allelic fraction
BGs: Background sample
cfDNA: Cell-free DNA
controls: Normal healthy cfdna samples
ctDNA: Circulating tumor DNA
FDR: False discovery rate
FFR: False family ratio
HWT: Wild-type cfdna control
NGS: Next generation sequencing
NSCLC: Non-small-cell lung carcinoma
PPV: Positive predictive value
RSDs: Reference standard samples
SNV: Single nucleotide variant
TMB: Tumor mutation burden
UMI: Unique molecular identifier
