# Supplementary figures and images for: A novel virtual barcode strategy for accurate panel-wide variant calling in circulating tumor DNA
Source: BMC Bioinformatics. 2020 Apr 3;21:127. doi: 10.1186/s12859-020-3412-2 (PMC7118954; doi:10.1186/s12859-020-3412-2)

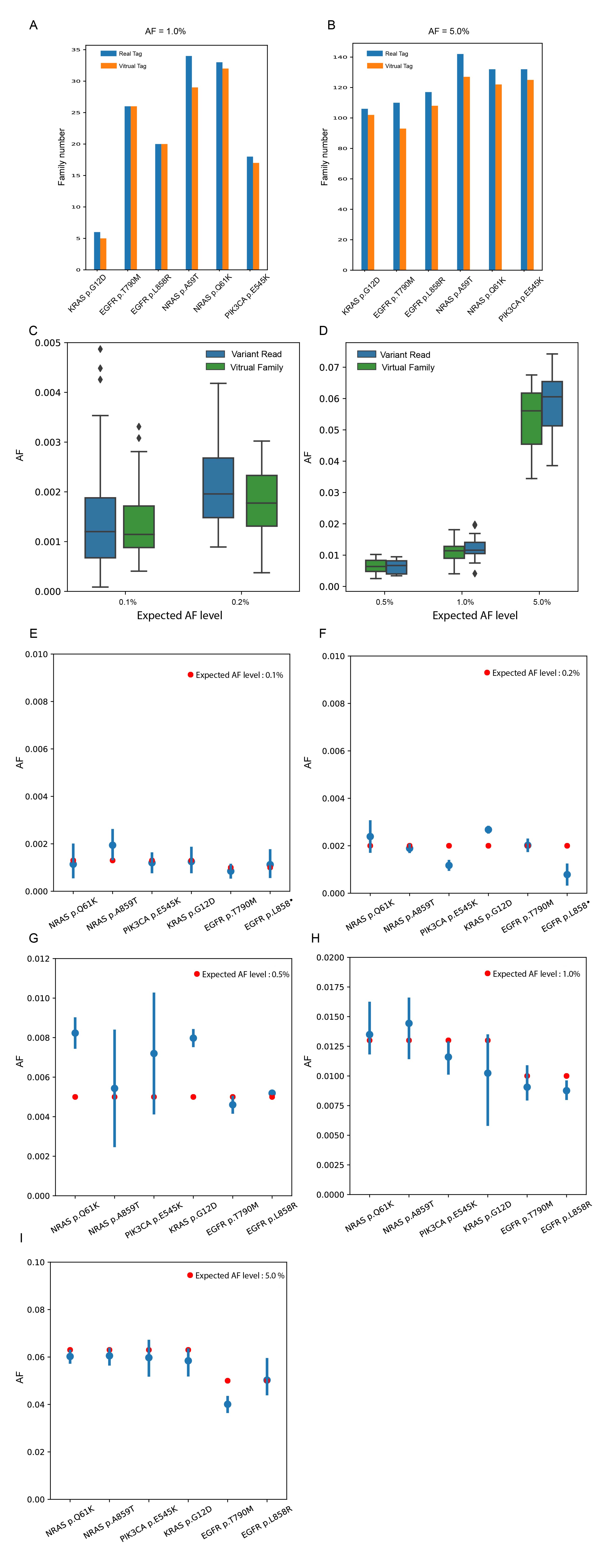

Supplement: Supplementary file 1 — Additional file 1. Figure S1. Non-reference template numbers and AF values calculated from different template features for six positive sites in RSDs. [file 12859_2020_3412_MOESM1_ESM.jpg]

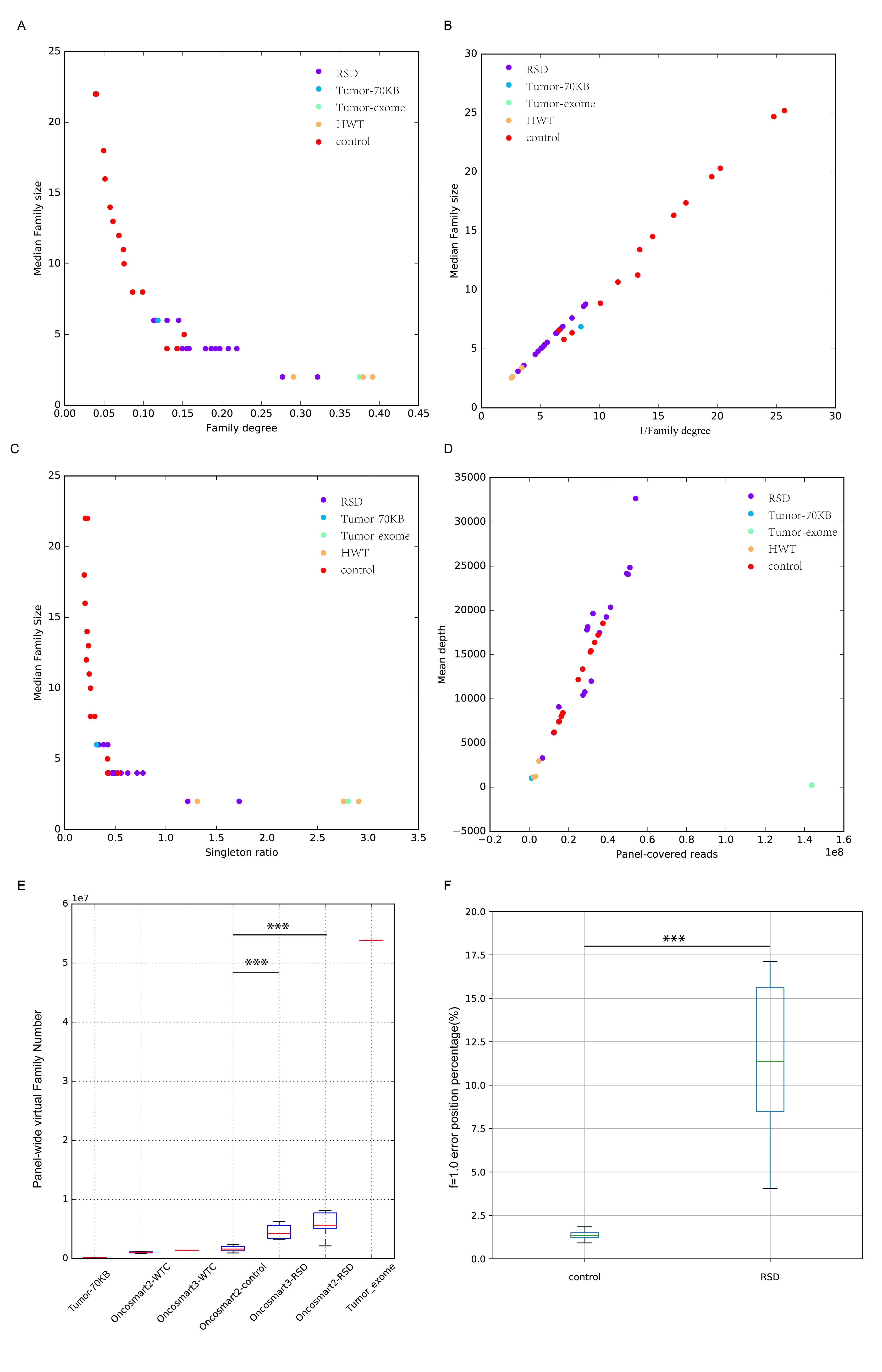

Supplement: Supplementary file 2 — Additional file 2. Figure S2. Relationships among virtual family degree, singleton ratio and median virtual family size, covered reads and sequencing depth among thirty BGs, three HWTs, and two tumor samples panel-widely. [file 12859_2020_3412_MOESM2_ESM.jpg]

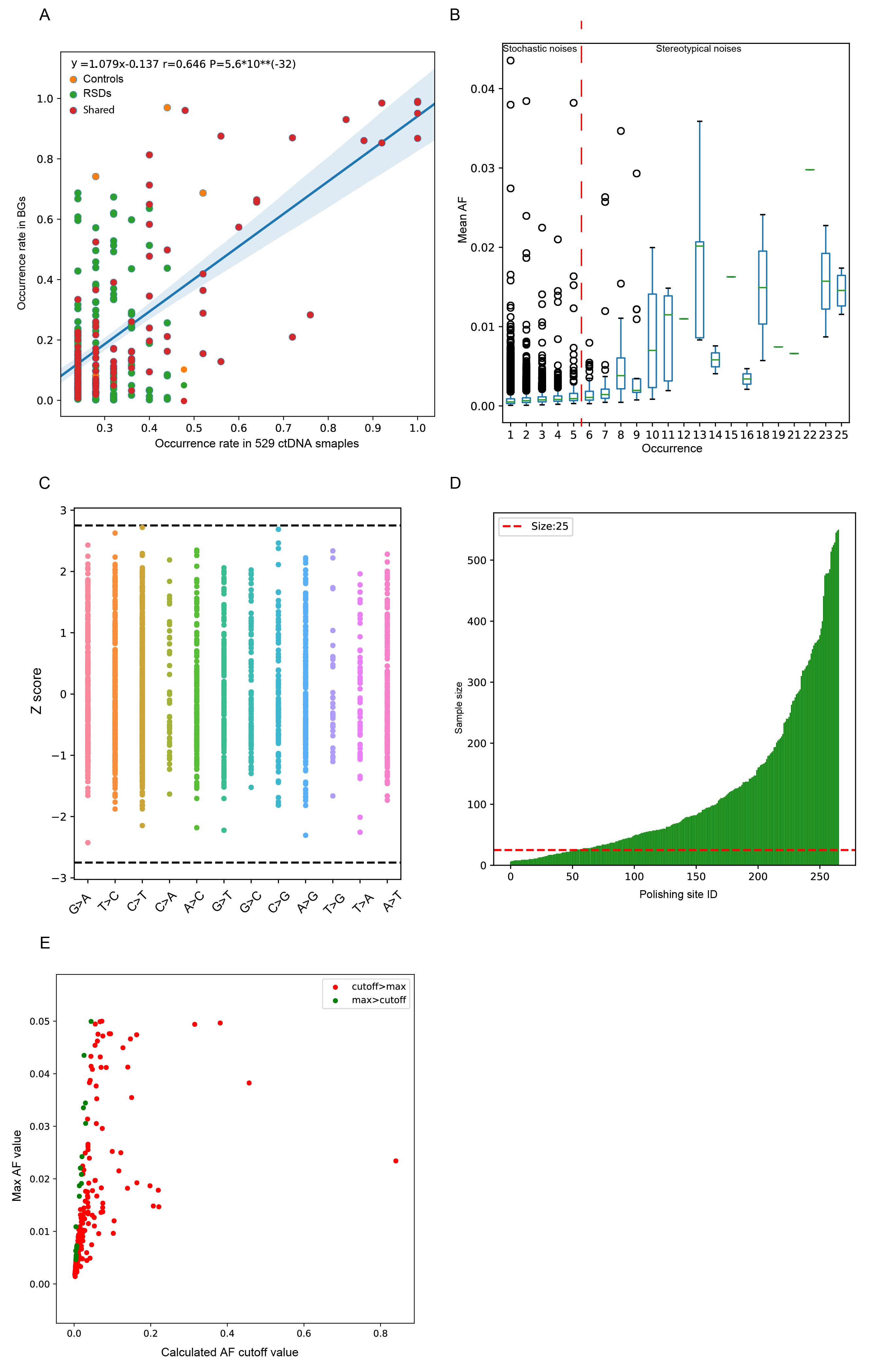

Supplement: Supplementary file 3 — Additional file 3. Figure S3. Characteristics of mutant-family-level noises. [file 12859_2020_3412_MOESM3_ESM.jpg]

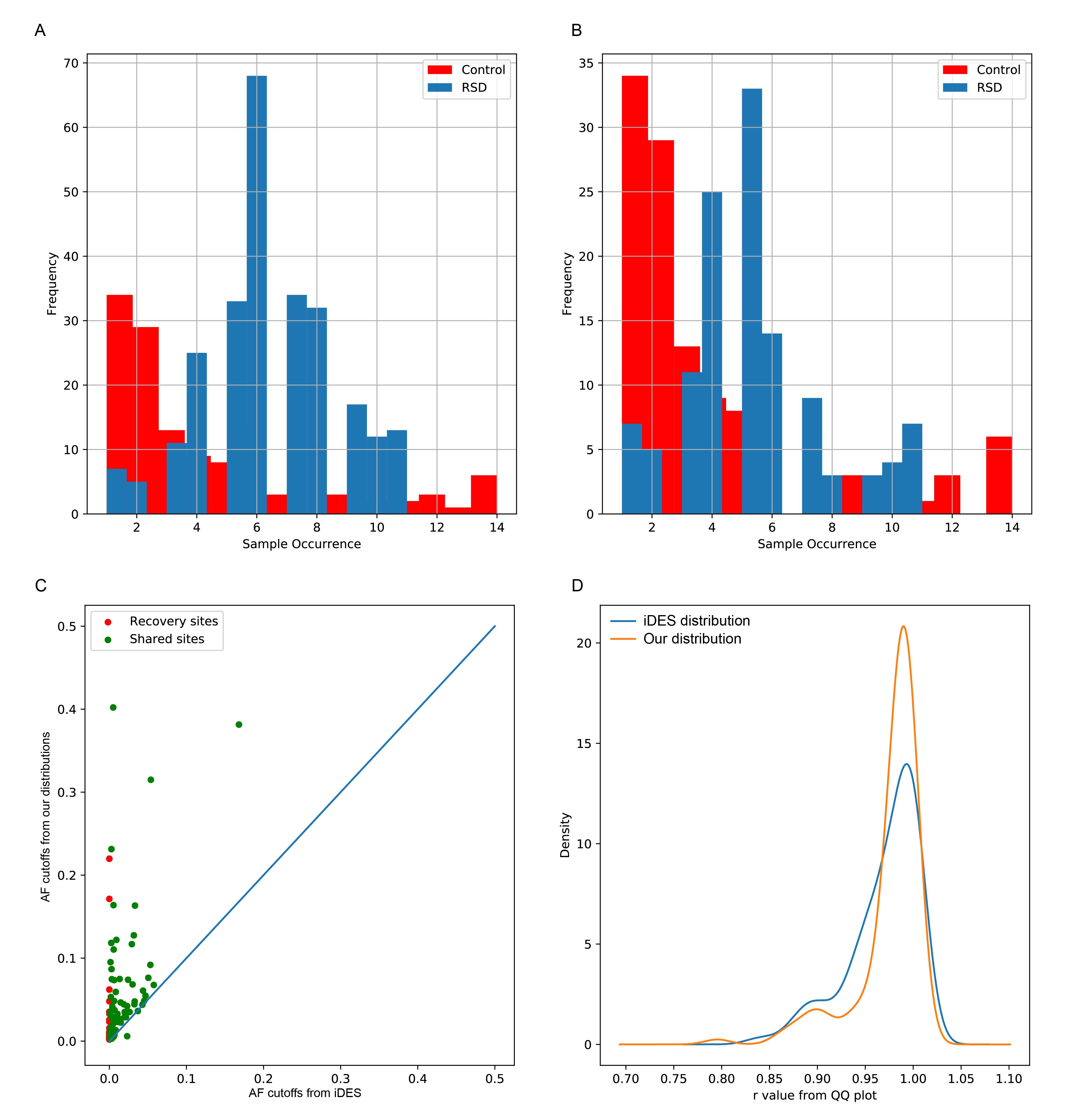

Supplement: Supplementary file 4 — Additional file 4. Figure S4. Sources of polishing sites and comparisons with iDES. [file 12859_2020_3412_MOESM4_ESM.jpg]

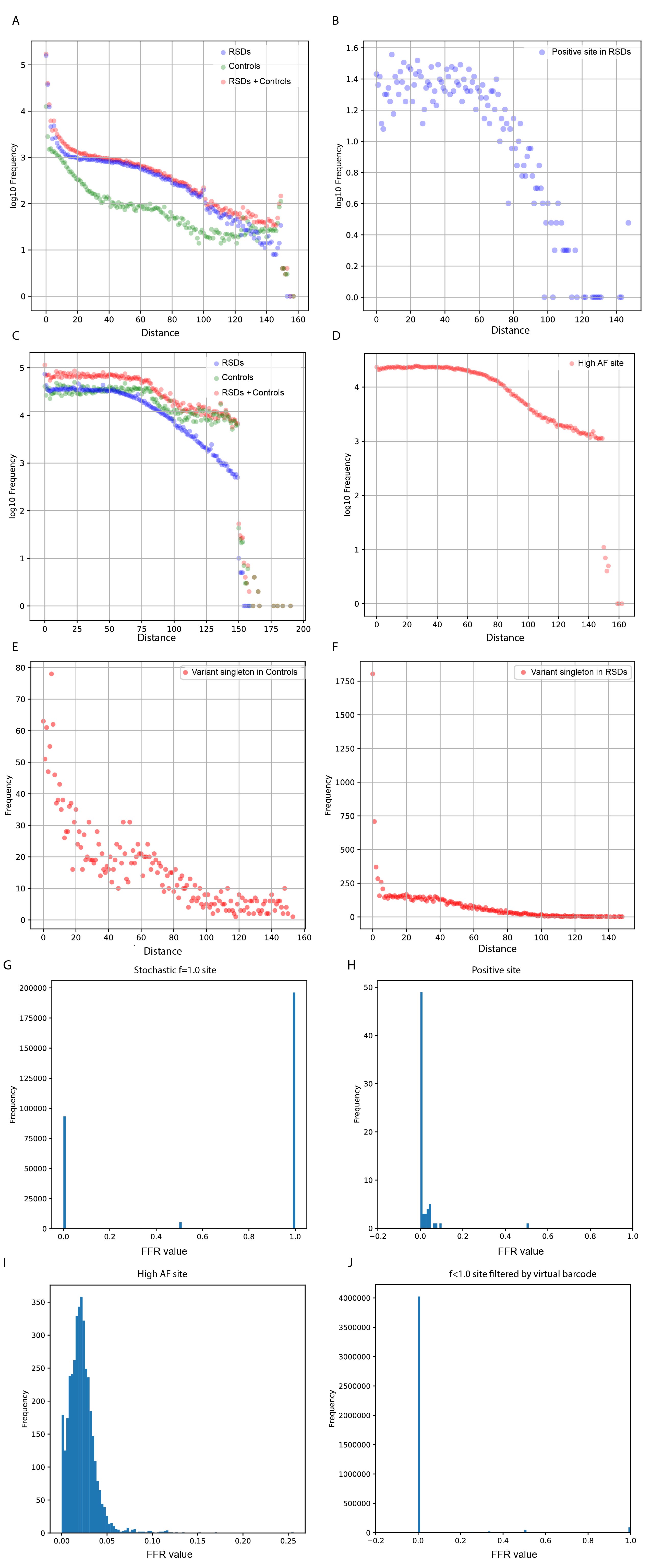

Supplement: Supplementary file 5 — Additional file 5. Figure S5. Distance distributions (Ds) and false family ratio (FFR) distributions at four kinds of site and variant singletons. [file 12859_2020_3412_MOESM5_ESM.jpg]

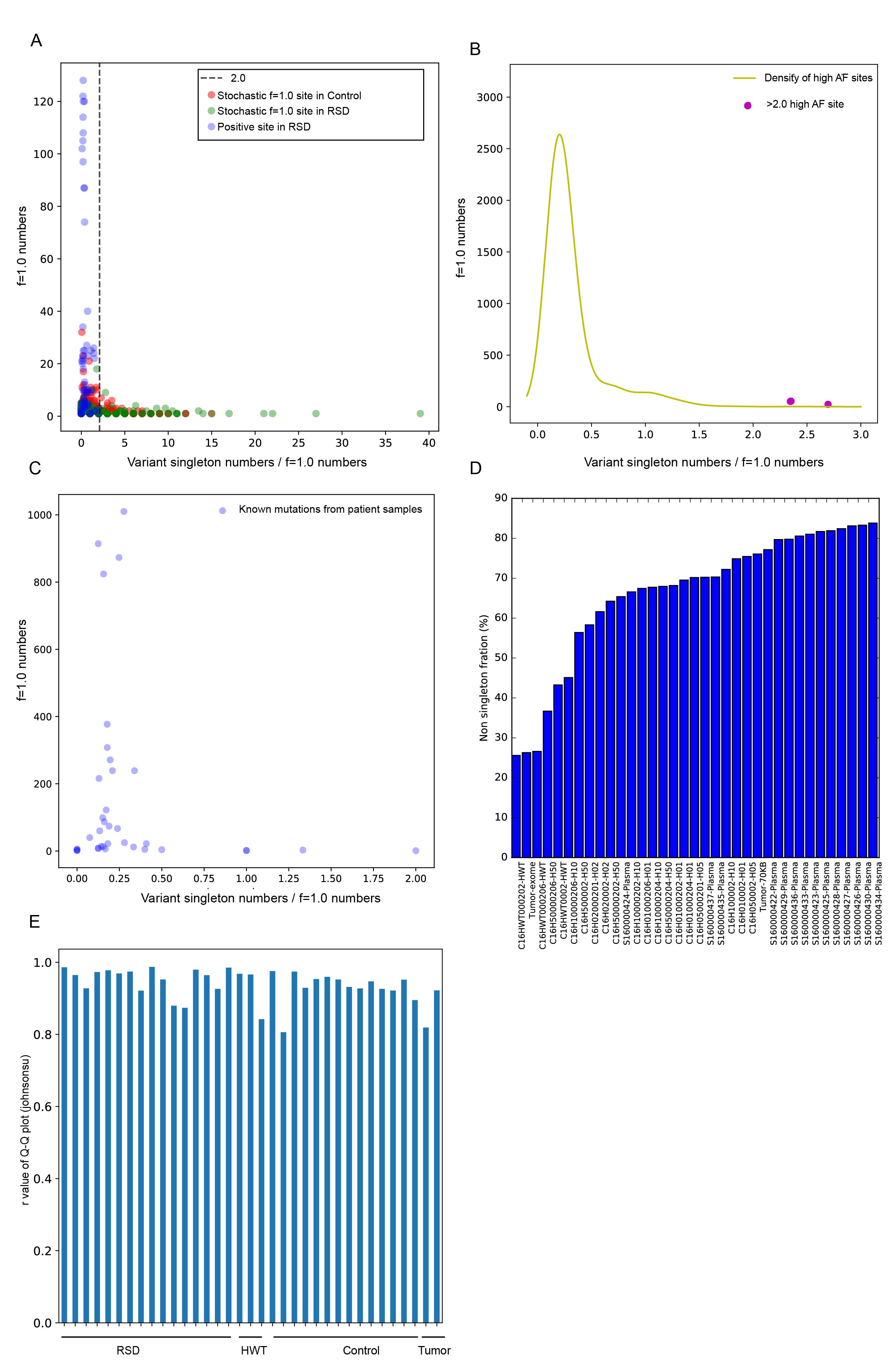

Supplement: Supplementary file 6 — Additional file 6. Figure S6. Imbalanced variant singleton ratios. [file 12859_2020_3412_MOESM6_ESM.jpg]

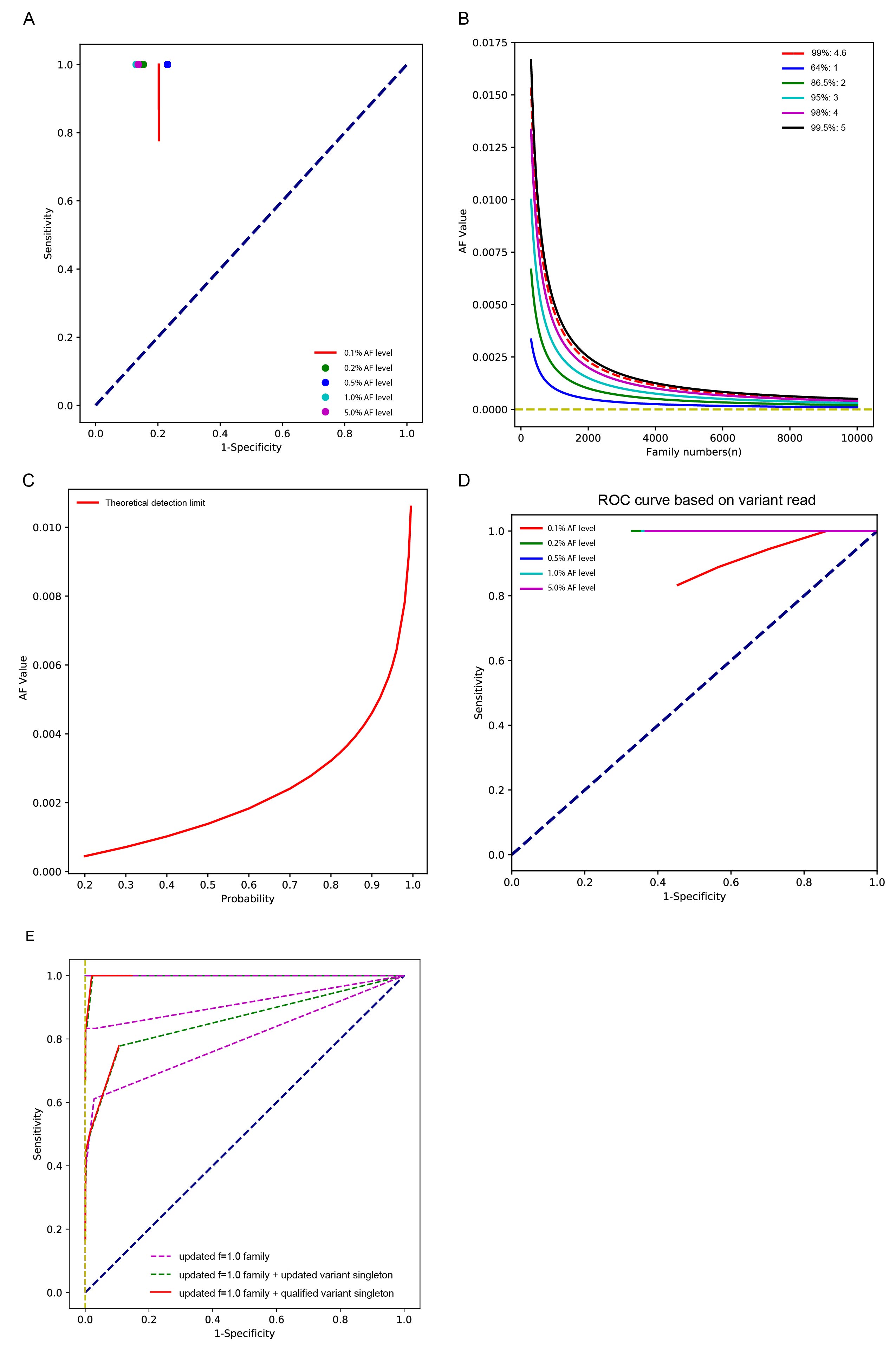

Supplement: Supplementary file 7 — Additional file 7. Figure S7. Theoretical detection limit and ROC curve based on different template features. [file 12859_2020_3412_MOESM7_ESM.jpg]

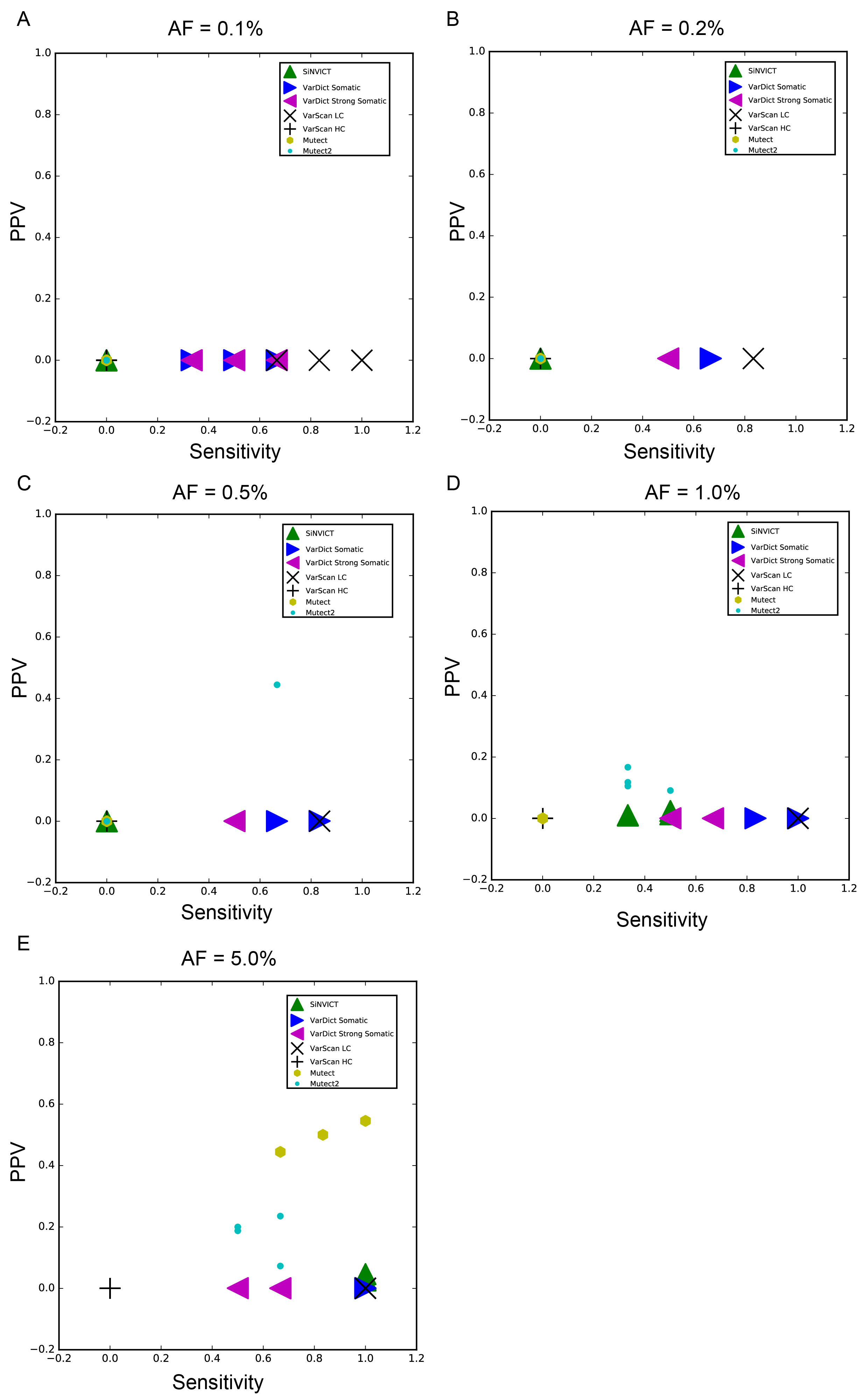

Supplement: Supplementary file 8 — Additional file 8. Figure S8. Sensitivity and PPV in relation to AF values from 0.1% to 5% in case of Mutect (yellow circle), Mutect2 (green circle), SiNVICT (green triangle), VarScan2 (Low confidence: black cross; High confidence: black plus), VarDict (blue triangle: somatic; purple triangle: strong somatic). [file 12859_2020_3412_MOESM8_ESM.jpg]

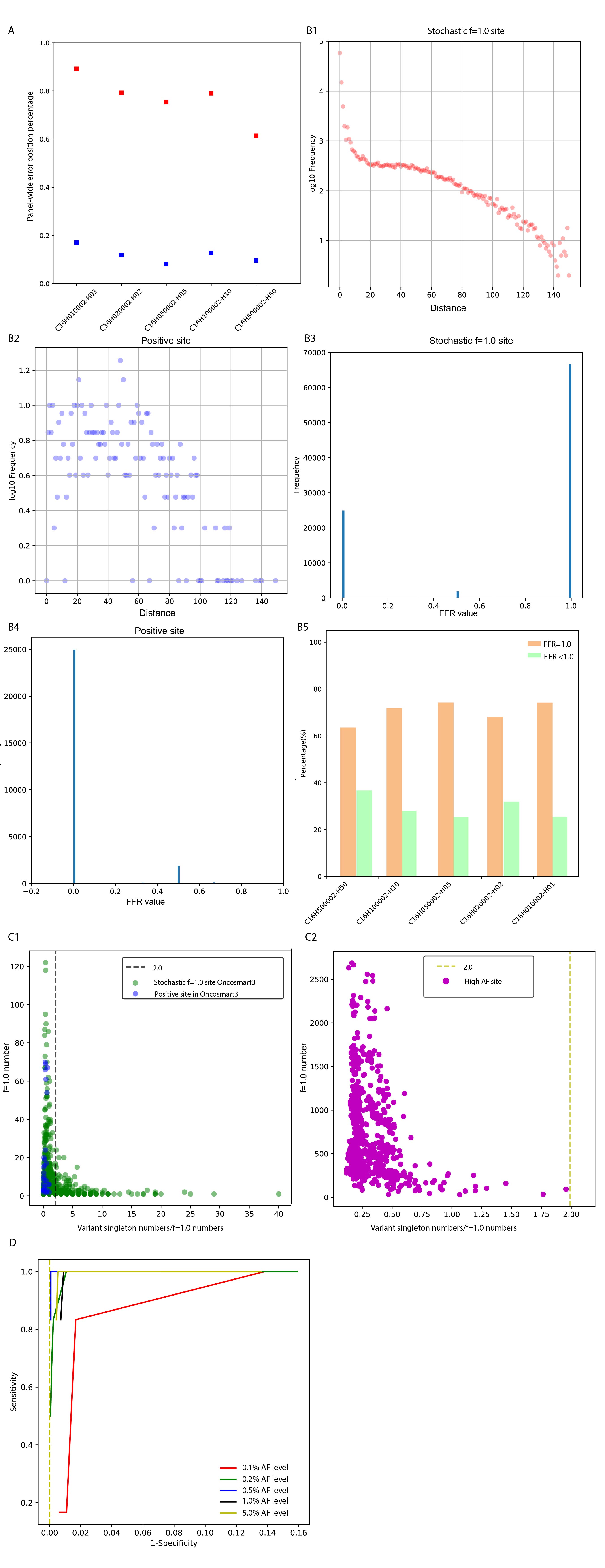

Supplement: Supplementary file 9 — Additional file 9. Figure S9. Validation of every filter using 5 Oncosmart3 RSDs. [file 12859_2020_3412_MOESM9_ESM.jpg]
