# Supplementary material for: A novel virtual barcode strategy for accurate panel-wide variant calling in circulating tumor DNA
Source: BMC Bioinformatics. 2020 Apr 3;21:127. doi: 10.1186/s12859-020-3412-2 (PMC7118954; doi:10.1186/s12859-020-3412-2)
Supplement: Supplementary file 16 — Additional file 16. Supplementary methods. [file 12859_2020_3412_MOESM16_ESM.docx]

A novel virtual barcode strategy for accurate panel-wide variant calling in circulating tumor DNA

Leilei Wu^1^, Qinfang Deng^5^, Ze Xu^3^, Songwen Zhou^5*^, Chao Li^2, 3*^, Yi-Xue Li^1, 2, 4*^

***1****. School of Life Sciences and Biotechnology, Shanghai Jiao Tong University, Shanghai, 200240, China;*

***2****. Shanghai Center for Bioinformation Technology, Shanghai, 201203, China;*

***3****. Smartquerier Biomedicine, Shanghai, 201203, China;*

***4****. CAS Key Laboratory of Computational Biology, CAS-MPG Partner Institute for Computational Biology, Shanghai Institute of Nutrition and Health, Shanghai Institutes for Biological Sciences, University of Chinese Academy of Sciences, Chinese Academy of Sciences, Shanghai, 200031, China;*

***5****. Department of Oncology, Shanghai Pulmonary Hospital, Tongji University School of Medicine, Shanghai, 200433, China.*

******* Corresponding authors.

Email addresses: songwenzhou2017@vip.126.com (Songwen Zhou), [lichao@smartquerier.com](mailto:lichao@smartquerier.com) (Chao Li), [yxli@sibs.ac.cn](mailto:yxli@sibs.ac.cn) (Yi-Xue Li)

**Supplementary Methods**

**Library preparation and sequencing**

16 cfDNA reference standard samples (RSDs) were ordered from Horizon Discovery Company. Hybrid selections were performed using three customized SeqCap EZ Choice Library, namely, Oncosmart1, Oncosmart2 and Oncosmart3, all of which covered important cancer driver genes and actionable hotspots. 14 normal health cfDNA controls and 11 RSDs were enriched in Oncosmart2 and the rest 5 RSDs (0.1%, 0.2%, 0.5%, 1% and 5%) were enriched in Oncosmart3. These 30 samples were our background samples (BGs). Three of 11 Oncosmart2 RSDs were UMI samples with 9-mer nucleotide tag added to single-strand DNA at AF level: 0.1%, 1%, and 5%.

Indexed Illumina next generation sequencing (NGS) libraries were prepared from plasma DNA and germline genomic DNA. For plasma DNA, 20-30 ng of DNA was used for library construction without additional fragmentation. An NGS library was prepared using the KAPA library preparation kit (Kapa Biosystems). Agencourt AMPure XP beads (Beckman-Coulter) were used to purify the extracted DNA. A 100-fold mole excess of ligation Illumina TruSeq adaptors were used for ligation at 16℃ for 16 hours. By adding 40 μL (0.8X) PEG buffer to enrich the ligated DNA fragments, a single step size selection of DNA fragments was performed. The ligated fragments were amplified using 500 nM Illumina backbone oligonucleotides, and 4-9 PCR cycles were assigned based on the input DNA mass.

The library concentration was assessed by Qubit and Qpcr. The fragment length was determined on a 2100 bioanalyzer using the DNA 1000 kit (Agilent). The library used a customized NimbleGen SeqCap EZ Choice Library for hybridization selection. Eight and 10 indexed Illumina libraries were included in single capture hybridization. After hybridization selection, the captured DNA fragments were mixed with 50 μL KAPA HiFi Hot Start Ready Mix (1X) and 2 μM Illumina backbone oligonucleotides. Then PCR was performed after 4 to 6 independent PCR with 12 to 14 cycles each. The PCR product was then pooled and treated with the QIAquick PCR Purification Kit (Qiagen). Finally, 2x150bp pair-end sequencing of the acquired multiple libraries was performed using Illumina HiSeq X10. For 3 UMI samples，2x100bp pair-end sequencing of acquired multiple libraries was performed using Illumina HiSeq 2500.

**Validation of the virtual tag/barcode and virtual-barcode based algorithm**

The detailed validation processes using three Oncosmart2 UMI samples were described as following three steps:

1) We randomly selected genomic positions on Oncosmart2 panel for 10 times (20,000 random positions per time). We clustered families by three tags: UMI only; Real tag: UMI, start site and template length; Virtual tag: start site, template length, and strand. Mean family number and standard deviation (SD) were calculated using ten samples. Subsequently, using 20,000 sites at one sample, a linear regression was constructed between Np and Nr (Np: virtual family numbers; Nr: real family numbers), and recovery rate for real family numbers was calculated as Np/Nr*100.

2) Family contents were compared between real families and virtual families at 20,000 sites. A few of virtual families contains different real families. These virtual families were classified by real family numbers, the percentages of which were calculated subsequently.

3) Different efficiencies of the virtual tag and real tag on decreasing noises were explored using following procedures:

3.1) For every family j at every genomic site i, we enumerated all reads: Fji and variant reads: Vji supporting a non-reference allele. Ratio of non-reference allele (f value) for every family was calculated as Vji/Fji.

3.2) Set f=1.0 in present study and eliminate genomic sites without f=1.0 family supports.

3.3) All left sites were used to calculate panel-wide error rate and panel-wide error position percentage (see **Panel-wide error rate and panel-wide error position percentage**). Their mean and SD were calculated to measure the effectiveness of decreasing noise in three UMI RSDs.

After validation, we further defined virtual barcode/tag as the start and template length. For every virtual family with a size larger than 2 at a specific position, f value and family strand constitution were recorded. For every virtual family, if a read was read1 (R1) and not reverse-complemented, or a read was read2 (R2) and reverse-complemented, the read originated from the plus (‘+’) strand. Otherwise, the read originated from the minus (‘–‘) strand. The set of all strands in a family represented the family strand constitution. The virtual family with virtual duplexes was harboring both ‘–‘and ‘+’ strands.

AF values at the virtual-family level were calculated only for positions with at least one f=1.0 virtual family support in a format: (Nra+Ns)/(F2+Ns) (Nra: the f=1.0 family number; Ns: the number of variant singleton; F2: the number of family with sizes larger than 2). AF values at the variant-read level were calculated for positions as: Nvariant/Ntotal (Nvariant: variant read numbers; Ntotal: total read numbers).

**Preprocessing**

Pair-end reads were aligned to the hg19 reference genome using the BWA (V0.7.15-r1140)-mem ([1](#_ENREF_1)) command, then sorted and indexed using SAMtools ([2](#_ENREF_2)). For every sample, Vardict([3](#_ENREF_3)) was used to do pileup for positions in the panel regardless of the non-reference allele frequency. Then variant candidates were generated. Genomic position, chromosome, reference base and non-reference allele of variant candidates were recorded. Germline variant sites and sites with low sequencing depth (<=1000) were excluded from candidates. Reads with low base qualities (<Q30) and mapping qualities (<30) were removed.

An in-house python script was used to evaluate the various statistics in the panel, including mapping statistics, virtual family degree, median virtual family size and singleton ratio (Supplementary Table S1). Here, the virtual family degree of the panel was defined as Nvf/Nread, (Nvf: number of virtual family; Nread: number of qualified reads). From definition, the virtual family degree was a statistic reflecting the ratio of DNA templates (virtual family) to covered reads in the whole panel. Singleton ratio of the panel was defined as (Ns<=2)/(Ns>2) (Ns<=2: virtual family size <= 2; Ns>2: virtual family size >2.).

**Construction of the polishing distribution** **and comparison with iDES**

Before selecting best-fit distribution for polishing sites, we did appropriate exploration work for best-fitted distributions of low-AF errors. After excluding COSIMIC and germline variant sites, we randomly sampled 1000 sites for 10 times using 529 Oncosmart2 cell-free DNA (cfDNA) data. All non-zero AFs were kept in one dimension vector: Pv. To mitigate the impact of outliers on over fitting, the largest AF value in the Pv was removed. Pv was fitted by 82 distribution iteratively. Four statistics were calculated: AIC, BIC, SEE, R from quantile-quantile plot (QQ-plot). Three main distributions that were ‘nct’, ’johnsonsu’ , ‘expnorm’ covered 75% ± 2*3.6% of sites . ‘Johnsonsu’ distribution was the most popular distribution based on the median of AIC, BIC, SEE and R (Table S3-1: Statistics of distributions). These results were validated independently using 104 Oncosmart1 patient samples (Table S3-2: Distributions from Oncosmart1). Based on the result of exploration and prior knowledge ([4](#_ENREF_4)), 10 distributions were selected as candidate distributions: ‘nct’, ’johnsonsu’, ‘expnorm’, ‘beta’, ‘gamma’, ‘norm’, ’weibull_min’, ‘dweibull’, ’lognorm’, ‘alpha’, which gave us a comprehensive distribution profile and demonstrated different site with different best-fit distribution.

Subsequently, personalized distribution was constructed at every polishing site. Due to the limited sample size of BGs, there was not enough statistic power to support one of candidate distributions. After validating stable occurrence rates for polishing sites (see **Polishing sites selection and occurrence rate validation** section), we recorded AF values in one-dimension vector with f=1.0 virtual family supporting both from 25 Oncosmart2 BGs and 529 Oncosmart2 cfDNA samples. This procedure increased sample size dramatically for most polishing sites (Figure S3D: 79.69% sites with sample size larger than 25). AF values in the vector were fitted by ten candidate distributions. Best distribution was selected based on AIC, BIC, SEE, r. AF cutoff value was adjusted using a stringent Bonferroni correction (where n = verctor size). Result showed that calculated AF cutoff values from 93.2% sites were larger than max AF value in the vector (Figure S3E). Left 6.8% (18 sites) showed that max AF values in the vector were larger than calculated AF cutoff values (Figure S3E: green dot). Here we adapted following strategies to set final AF cutoff values. If max AF value sin the vector was from our BGs and was larger than calculated AF cutoff value, then cutoff values were the max AF value. Otherwise, cutoff values were calculated values.

Second, we made a comparison with the iDES proposed polishing method. In iDES, only normal health cfDNA controls were applied for background construction. 81.9% of the 265 sites had a proper fit, but no distributions were available for the left 28.1% (Figure S4C: red dots), which was only happened one time in controls. In our method, based on the validation of stable occurrence rate of polishing sites, we used both 11 Oncosmart2 RSDs and 529 Oncosmart2 patient cfDNA samples to significantly increase the sample size (Figure S3D). This endeavor brought three advantages over iDES. First, all the sites were fitted to distributions. Second, our method exhibited a higher overall site fit efficiency than iDES. The density plot of the R value from the fitted Q-Q plot showed that our method had a significantly higher peak than iDES (Figure S4D). This improvement was due to the proper selection of candidate distributions and details are provided in Table S3. Third, we believe that cutoff values from the large sample size-based distributions are more reasonable and robust than values from small size-based distributions. Nearly all AF cutoff values obtained using our method were greater than the corresponding values obtained from iDES (Figure S4C).

**Stereotypical noise selection and corresponding characteristics**

We grouped mutant-family-level noise by genomic position and substitution type among 25 Oncosmart2 BGs. Based on the relation between occurrence and AF spectra, we selected 265 polishing candidates and recoded their sample occurrence in a vector Pg. In 529 cfDNA samples, sample occurrence of every candidate with at least one f=1.0 family support was recorded in vector Pc. Then occurrence rate was calculated as Pg/25 for Oncosmart2 BGs and Pc/529 for Oncosmart2 cfDNA samples. Linear relationship between Pg/25 and Pc/529 was explored for occurrence rate validation.

**Polishing sites substitution analysis**

Percentages of 12 base substitution types for 265 polishing site were calculated. For every substitution type, Z score of AF was calculated. In detail, mean AF (AFm) and SD (AFsd) were obtained for every substitution type. In every substitution type, Z score of single sample AF (AFs) was calculated as (AFs – Afm)/AFsd. Z score value spectra for 265 polishing sites were used to demonstrate the stability of AF values.

**Additional fine-tuning filters**

**1. Variant position in a segment**

For every virtual family, genomic position of variant was obtained. Distances between variant genomic position and virtual family’s start as well as end were calculated using following formula：D1 = Pc – Ps; D2 = Pe - Ps; D1, D2 were distances of the variant position to the start and end at the virtual family respectively. Pc, Ps and Pe were genomic positions of variant, start and end of the virtual family. The minimum of D1 and D2 was obtained as the absolute distance (Ds value) of variant in a virtual family.

Ds value of every f=1.0 virtual family at high-AF site, positive site, stochastic f=1.0 site was calculated. Also Ds value of every f<1.0 virtual family at genomic site filtered by virtual barcode step was calculated. Ds distributions were constructed for these 4 kinds of site. Additionally we calculated Ds and constructed Ds distribution for variant singleton. Based on Ds distributions, specific Ds values (<=2 and >=149) at stochastic f=1.0 site were set in present study, and f=1.0 virtual family with specific Ds values was regarded as false family. For every site, false family ratio (FFR) that was the false family numbers to all f=1.0 family numbers was calculated. (Details are provided in **False family ratio calculation at genomic site level** section). This ratio clearly demonstrated impact of false family in every BG.

Finally we applied this customized filter to remove false families and false variant singletons. And in turn, we updated f=1.0 family numbers as well as variant singleton numbers and removed sites without updated f=1.0 virtual family support.

**2. Imbalanced singleton number**

At the panel level, panel-wide singleton ratios were calculated for all BGs, 3 HWTs, and 2 tumor samples (Details in **Preprocessing)**.

At genomic site level, singleton ratio was defined as Ns/Nra (Ns: updated variant singleton numbers; Nra: updated f=1.0 virtual family numbers). Scatterplot between singleton ratio and f=1.0 family numbers was plot for positive site, stochastic f=1.0 site, high-AF site. Appropriate singleton ratio (2.0) was selected to avoid over-recovery of variant singletons. This singleton ratio was validated in longitudinal cfDNA samples from 53 NSCLC patient with known somatic mutations from our previous work ([5](#_ENREF_5)).

At sample level, singleton ratio values were stable at genomic variant site with high AF value (Figure S6B; Figure S9C2) and positive/known variants (Figure S6A,C, Figure S9C1: blue dots). Extreme high singleton ratios appeared at low-AF genomic variant site (Figure S6A, Figure S9C1: read and green dots). No correlations were between panel-wide singleton value and mean variant singleton value among BGs. However, significant and perfect linear relationship were showed between panel-wide singleton ratios and mean variant singleton ratios at sites with AF>=0.05. Based on this observation, we concluded that at real variant site, variant singleton ratio fluctuated around corresponding panel-wide singleton ratio. Thus, distribution of variant singleton ratios from AF>=0.05 sites were used as a background. Like AF distribution at polishing site, this ratios fitted well on ‘Johnsonsu’ distribution for every sample (Figure S6E). P value of every stochastic f=1.0 site was obtained. Multiple testing corrections were via false discovery rate (FDR) estimation. FDR value was set 0.01 to remove sites with extreme high variant singleton ratios. Left sites were all with qualified variant singletons.

**3. Minimum template number requirement**

To calculate minimum template number at different confidence level, we used ‘Poisson’ distribution. Previous study applies ‘Poisson’ distribution to calculated probability of detecting ≥1 mutant circulating tumor DNA (ctDNA) molecules and detection limit based on k tumor reports (1. p = 1 – e^-nd^; 2. d = ln(1–p)/(–nk) ) ([4](#_ENREF_4)). Here, based on ‘Poisson’ formulas, minimum variant ctDNA templates needed to reach specific confidence level (CI) were calculated. Based on formula 1, at specific template depth and CI, minimum variant ctDNA templates were obtained to reach particular detection limit. Relation between detection limit and template depth at different CI was simulated based on formula: d= ln(1–p)/(–n). Simulated result demonstrated that detection limit (d value) was monotonically decreasing as an increase in template depth (Figure S7B). At different CI , simulated lines would not intersect. These two natures of this function demonstrated that at certain CI, detection limit was determined by the number of variant ctDNA template at a site, and theoretical ctDNA template was equal to -ln(1–p).

For panel-wide calling, sensitivity and positive predictive values (PPV) were all needed to balance. Only specific template feature based on relative clean panel could effectively do panel-wide calling. At theoretical detection limit, we assumed that template number of stochastic f=1.0 sites were lower than real SNVs. Based on this assumption, different template features: raw variant read, updated f=1.0 family number, updated f=1.0 family numbers plus updated variant singleton, updated f=1.0 family numbers plus qualified variant singleton and one strong auxiliary indicator that was virtual duplex were explored. Theoretical minimum ctDNA template was calculated at a series of CIs from 80% to 99.5%. ROC curves were built at all AF levels: 0.1%, 0.2%, 0.5%, 1%, 5%. Then we selected the best template feature on the basis of optimal tradeoff between sensitivity and PPV.

**Statistics for performance assessment**

Sensitivity, PPV, false positive rate (FPR) and F1 values were used to compare the performance of our algorithm and five published calling algorithms using 11 Oncosmart2 RSDs. False positive rate (FPR) was also used to reported FPRs in iDES([4](#_ENREF_4)) and ERASE-seq([6](#_ENREF_6)). For iDES, reported sensitivity and PPV from 29 known variants and 279 preselected negative controls (as opposed to all variants across the entire panel) were used to infer to FPR. Here Sensitivity=no.TP/(no.TP+no.FN); Specificity=no.TN/(no.TN+no.FP); PPV=no.TP/(no.TP+no.FP), F1=2/(1/Sensitivity+1/PPV); False positive rate=no.FP/(no.FP+no.TN) where no.TP was the number of true-positive calls, no.FP was the number of false-positive calls, no.FN was the number of false-negative calls and no.TN was the number of true-negative calls.

**Panel-wide error rate and panel-wide error position percentage**

Panel-wide error rate was defined as previous research([4](#_ENREF_4)) that was the number of all non-reference bases divided by all sequenced bases among all sites. Panel-wide error position percentage was calculated as number of left variant sites divided by all sites.

**False family ratio calculation at genomic site level**

False family ratio was calculated at four kinds of site: stochastic f=1.0 site, high-AF site, positive site, f<1.0 site filtered by virtual tag. For the first three kind of sites, false family ratio was calculated as FFR = Nff/Nr (Nff :false family numbers; Nr r:f=1.0 family numbers). For f<1.0 sites, false family ratio was calculated as FFR = Nff/Nv (where Nv: f<1.0 family numbers).

**Benchmarking of the available calling algorithm**

We benchmarked and assessed the detection limit of 5 available panel-wide calling algorithms: Mutect, Mutect2, Vardict, Varscan2, SINVICT ([3](#_ENREF_3), [7-10](#_ENREF_7)) using 16 RSDs (11 Oncosmart2 RSDs and 5 Oncosmart3 RSDs). For Vardict, Varscan2, as their manual recommended, we calculated these statistical at both two confidence level results. For Mutect and Mutect2, sequence data was input after standard preprocessing steps including marking duplicates, recalibration of base quality scores and local realignment. For SiNVICT, as request, all required preprocessing were performed before SNV calling. To measure their performance, sensitivity, PPV, FPR and F1 score were calculated (Formulas in Statistics for performance assessment). All benchmarking code was available at: https://github.com/zhaodalv/virtual-barcode-based-calling/tree/master/benchmark_code.

**Other statistical analysis**

For two-group statistical test, Mann-Whitney U test was applied. All analyses were performed using the Python 2.7.

**Supplementary result**

**Preprocessing statistics**

In preprocessing step, reads covered in panel, bases covered in panel, virtual family degree, singleton ratio and mean/median virtual family size were obtained (Supplementary Table 1). Among all data, tumor exome sample had the largest template numbers (Table S1: marked red). For another tumor sample from 70kb panel, it had the lowest template numbers (Table S1: marked green). As panel size increases, so did the covered virtual family numbers (Figure S2E). Median Oncosmart2 template numbers was higher than median Oncosmart3 template numbers in RSDs. In RSDs, virtual family numbers were significantly higher than corresponding HWTs (Figure S2E; P<0.0001).

Number of panel covered coverage (number of panel covered reads) showed a good linear relationship with mean panel depth (Figure S2D). Then template numbers were normalized based on panel coverage. Close relationships were showed among virtual family degree, singleton ratio and median virtual family size. Virtual family degree was inversely related with median family size and directly related with singleton ratio. Family degree of exome sample was 37.5% and median family size was 2.0 (Figure S2A), which indicated most templates were sequenced one time and consisted of R1 and R2 only. For 70kb panel, low template numbers at high coverage caused high family degree and high median family size (Figure S2A). Compared with RSDs, controls had fewer template numbers and similar panel coverage/depth. Thus low family degrees and high family size were achieved in controls (Figure S2B).

**Stereotypical noise selection and corresponding characteristics**

After virtual barcode, all mutant-family-level noises were grouped by the genomic position and the substitution type. Occurrence and AF values of unique mutant-family-level noises were recorded. AF spectra of every occurrence group were explored. Result showed that median AF value increased as an increase in occurrence among 25 BGs (Figure S3B). Error sites with sample occurrence lager than 5 had relative stable AF spectra and lager median AF value than error sites with number of occurrence less than 5 (Figure S3B). Stable AF characteristic of high occurrence error sites was further demonstrated among 12 substitution types with Z scores located within two standard deviations (Std) (Figure S3C; **Polishing sites substitution analysis**). Thus, sites with number of occurrence larger than 5 were selected as stereotypical sites.

**Distance distribution and effect of false families**

Ds value showed a rapid decrease in the first three positions and subsequent leveling off for f=1.0 virtual families at stochastic f=1.0 site both in 14 Oncosmart2 controls and 11 Oncosmart2 RSDs (Figure S5A). For variant singleton, the same Ds pattern was observed both in controls (Figure S5E) and RSDs (Figure S5F). However, gradual drop was observed at positive site (Figure S5B), high-AF site and at f<1.0 site (Figure S5C,D). These results demonstrated that positions near two ends of the virtual family was the specific feature for f=1.0 families at stochastic f=1.0 site (Figure S5A). This phenomenon might be caused by ignorance of phasing corrections for first 3 cycle at illumina XTM (https://support.illumina.com/content/dam/illumina-support/documents/documentation/system_documentation/hiseqx/hiseq-x-system-guide-1505009107.pdf). Previous study showed that errors happened at 3’ end of sequence with higher error rates([11](#_ENREF_11)), which might be caused by phasing in illumina ensemble-based sequencing-by-synthesis (SBS) ([12](#_ENREF_12)), which led to accumulating failures in incorporation of fluorescent dNTP at 3’ ends. Distributions among variant singletons showed no increasing trend at 3’ ends among variant singletons both in 14 Oncosmart2 controls (Figure S5E) and 11 Oncosmart2 RSDs (Figure S5F), which might be due to new empirical phasing correction algorithm. Contrary to Ds distributions in variant singleton, a suddenly decline at end in all families at different sites (Figure S5A, B, C, D), which indicated random base calls caused by phasing and prephasing were depressed by our virtual tag. Based on above analysis and results, we selected proper Ds values that were <=2 and >=149. Families with these Ds values were regarded as false families. Finally, the impact of false family was explored by calculating FFR at every genomic position (Details in **False family ratio calculation at genomic site level**). Result showed that a majority of stochastic f=1.0 sites were entirely consisted of false families (Figure S5G; FFR=1.0), while no variants with FFR=1.0 were found at positive site, high-AF site (Figure S5H, I) and a small proportion in f<1.0 site (Figure S5J).

**Selection of template features**

In present study, we used ‘Poisson’ distributions to calculated minim variant allele depth required at different CI (Details in **Additional fine-tuning filters section**). Based on stimulated data from ‘Poisson’ distribution, detection limit monotonically increased as the rise of CI (Figure S7C). A series of required template numbers at CIs from 0.8 to 0.995 were used to build ROC for different template features including raw variant read, updated f=1.0 family, updated f=1.0 family plus updated variant singleton and updated f=1.0 family plus qualified singleton. Raw variant read as a template feature achieved high sensitivity that was up to 1, but specificity was poor at every AF level (Figure S7D). This result also demonstrated that errors made the number of variant raw read not fit ‘Poisson’ distribution well at high depth (Figure S7B). Updated f=1.0 family number could effectively distinguish between true positive and false positive sites (Figure S7E, dash pink line). Updated f=1.0 family number plus qualified variant singleton was the most specific feature and had a largest AUC (Figure S7E, read line) at every AF level among all template features.

Besides, the role of virtual duplex in distinguishing real variants was also validated. The results showed that the virtual duplex could effectively recognize true positive sites at every AF level (Figure S7A), but was not a necessary condition for true positive sites, which indicated that the virtual duplex provided strong evidence for hotspot mutations and known mutations among longitudinal cfDNA data, as in a previous UMI-based study([4](#_ENREF_4)). For panel-wide calling, the virtual duplex could be an effective auxiliary indicator.

**Benchmarking of available algorithms at AF levels ranging from 0.1% to 5%**

Sixteen RSDs with AF levels ranging from 0.1% to 5% were used for benchmarking 5 available calling software. Results showed that in sensitivity aspect, SiNVICT detection limit was 1% and performed well at 5% level (Figure S8D, E: green triangle). Vardict could detect SNV at all AF level with mean sensitivity higher than 50% under both its somatic (Figure S8: blue triangle) and strong somatic condition (Figure S8: purple triangle). Somatic mutations called by Varscan2 under low confidence condition (Figure S8: black cross) had high mean sensitivity value at all AF levels. However, none of these were regarded as high confident mutations (Figure S8: black plus). For Mutect, detection limit was 5% (Figure S8E: yellow circle). Although detection limit of Mutect2 reached 0.5%, its performance was unstable among samples at this level (Figure S8C: light green circle). In PPV aspect, Mutect and Mutect2 significantly outperformed the rest software from 0.5% to 5%. However, their mean PPV was still below 50%. At 5%, Mutect got the highest mean F1 score: 67.9%. However, maximum F1 among 5% samples was below 75% (62.5%~70.6%). More detailed data are provided in Table S4. The poor performance of published algorithms with AF from 0.1% to 5% indicated that they were not suited for detecting SNV with low number of variant ctDNA templates in the blood. Based on result of benchmarking, the development of novel calling algorithm accurate calling of low AF mutations based on high-sequencing-depth data was of great importance and necessity.

**Supplementary Figures, Tables**

Figure S1. Non-reference template numbers and AF values calculated from different template features for six positive sites in RSDs. (A) f=1.0 virtual family numbers (orange bar) and f=1.0 real family numbers (blue bar) in one UMI sample at 1.0 % AF level (B) f=1.0 virtual family numbers (orange bar) and f=1.0 real family numbers (blue bar) in one UMI sample at 5% AF level (C,D) Boxplot of AF spectra of six positive sites among samples at expected AF level ranging from 0.1% to 5%. Blue boxplot representing AF value calculated from variant read level; Green boxplot representing AF value calculated from virtual family level (E ~ I) AF spectra calculated from virtual family level at every positive site among samples with expected AF level from 0.1% to 5%. Read dot representing expected AF level for each positive site.

Figure S2: Relationships among virtual family degree, singleton ratio and median virtual family size, covered reads and sequencing depth among thirty BGs, three HWTs, and two tumor samples panel-widely. (A) Inverse relationship between family degree and median family size. (B) Significant linear relationship between the reciprocal of family degree and median family size. (C) Inverse relationship between Singleton ratio and median family size. (D) Linear relationship between panel-wide covered reads and sequencing depth. (E) Boxplot of panel-wide virtual family numbers among different panels, significantly higher virtual family numbers in RSDs than controls and corresponding HWTs (***means P<0.001). (F) Boxplot of f=1.0 error position percentage between controls and RSDs (***means P<0.001).

Figure S3: Characteristics of mutant-family-level noises. (A) Scatter plot of occurrence rates for 265 polishing sites between 25 Oncosmart2 BGs and 529 cfDNA samples (blue dot: polishing site only in 14 Oncosmart2 controls; orange dot: polishing site only in 11 Oncosmart2 RSDs; green dot: shared polishing sites between Oncosmart2 controls and RSDs). (B) For every unique mutant-family-level noise, boxplot of AF spectra at every occurrence among 25 BGs . (C) Z score profiles for 265 polishing sites at every substitution type (horizontal black dotted line representing Z score value for P=0.01). (D) Distribution of ranked sample size for 265 polishing sites among 25 Oncosmart2 BGs and 529 cfDNA samples. (E) Calculated AF cutoff values from best fitted distributions and max AF values from recorded AF values in one-dimension verctor with f=1.0 virtual family supporting from 25 Oncosmart2 BGs and 529 Oncosmart2 cfDNA samples; Red dot representing calculated AF cutoff value larger than max AF value; green dot representing calculated AF cutoff value less than max AF value.

Figure S4. Sources of polishing sites and comparisons with iDES. (A) Sample occurrence distributions for 265 polishing sites among 14 Oncosmart2 controls and 11 Oncosmart2 RSDs (red bar represents controls and blue bar represents RSDs). (B) Sample Occurrence distributions for 121-shared polishing sites among 14 Oncosmart2 controls and 11 Oncosmart2 RSDs (red bar represents controls and blue bar represents RSDs). (C) Relation between cutoff values calculated from iDES distributions and our distributions for 87 shared sites (green dots). Red dots representing recovered 34 AF cutoffs calculated only from our distribution while ‘NA’ from iDES. (D) Density of R value for iDES (blue line) and our distributions (orange line) for 87 and 121 shared sites respectively.

Figure S5. Distance distributions (Ds) and false family ratio (FFR) distributions at four kinds of site and variant singletons. (A) Ds distributions of f=1.0 virtual families at stochastic f=1.0 site from 11 Oncosmart2 RSDs (blue dot), controls (green dot) as well as 25 BGs (red dot) respectively. (B) Ds distributions of f=1.0 virtual families at positive site from 11 Oncosmart2 RSDs. (C) Ds distributions of f<1.0 virtual families at genomic site filtered by virtual tag from 11 Oncosmart2 RSDs (blue dot), controls (green dot) as well as 25 BGs (red dot) respectively. (D) Ds distributions of f=1.0 virtual families at high-AF site from 25 BGs. (E) Ds distributions of variant singletons at stochastic site from 14 Oncosmart2 controls. (F) Ds distributions of variant singletons at stochastic f=1.0 site from 11 Oncosmart2 RSDs. (G) False family ratio distribution at stochastic f=1.0 site from 25 BGs. (H) False family ratio distribution at positive site from 11 Oncosmart2 RSDs. (I) False family ratio distribution at high-AF site from 25 BGs. (J) False family ratio distribution at genomic site filtered by virtual tag from from 25 BGs.

Figure S6: Imbalanced variant singleton ratios. (A) Scatterplot between updated f=1.0 family numbers and corresponding ratio of updated variant singletons to updated f=1.0 virtual family numbers at stochastic f=1.0 site in Oncosmart2 RSDs (green dot), Oncosmart2 controls (red dot), and at six positive site from Oncosmart2 RSDs at every AF level (blue dot) (the black vertical dotted line representing a singleton ratio of 2.0). (B) Density curve between updated f=1.0 family numbers and corresponding ratio of updated variant singletons to updated f=1.0 virtual family numbers among all high-AF sites in 25 Oncosmart2 BGs. (C) Scatterplot between updated f=1.0 family numbers and corresponding ratio of updated variant singletons to updated f=1.0 virtual family numbers among known somatic mutation sites from longitudinal cfDNA data from 53 NSCLC patients^(^[^5^](#_ENREF_5)^)^. (D) Fraction of non-singletons at every BG sample. (E) R value of Q-Q plot from fitted ‘Johnsonsu’ distribution for variant singleton ratio at high-AF (>=0.05) site for every BG sample.

Figure S7. Theoretical detection limit and ROC curve based on different template features. (A) ROC curve constructed based on a combination of virtual duplex and non-reference allele template numbers. (B) Simulated theoretical minimum AF (detection limit) at different depth of template for a particular confident level (read dot line: 99% confident level). (C) Simulated theoretical minimum AF at different confident level. (D) ROC curve constructed from variant reads. (E) ROC curve constructed from different template features.

Figure S8. Sensitivity and PPV in relation to AF values from 0.1% to 5% in case of Mutect (yellow circle), Mutect2 (green circle), SiNVICT (green triangle), VarScan2 (Low confidence: black cross; High confidence: black plus), VarDict (blue triangle: somatic; purple triangle: strong somatic).

Figure S9. Validation of every filter using 5 Oncosmart3 RSDs. (A) Panel-wide error position percentage in every Oncosmart3 RSDs before (read square) and after virtual tag (blue square) (B1). Ds distributions of f=1.0 virtual families at stochastic f=1.0 site from Oncosmart3 RSDs (B2) Ds distributions of f=1.0 virtual families at positive site from Oncosmart3 RSDs. (B3) False family ratio(FFR) distribution at genomic site with f=1.0 (B4) False family ratio(FFR) distribution at positive site (B5) Fraction of variants totally consisted with false family (orange bar) at stochastic f=1.0 site for Oncosmart3 RSDs (C1) Scatter plot between updated f=1.0 family numbers and corresponding ratio of updated variant singletons to updated f=1.0 virtual family numbers at stochastic f=1.0 site from Oncosmart3 RSDs (green dot), and at positive site (blue dot) (black vertical dot line representing singleton ratio =2.0). (C2) Scatter plot between updated f=1.0 family numbers and corresponding ratio of updated variant singletons to updated f=1.0 virtual family numbers at high-AF site from Oncosmart3 RSDs (yellow vertical dot line representing singleton ratio =2.0) (D) ROC curve based on optimal template feature (updated f=1.0 virtual family plus qualified variant singleton) at every AF level with theoretical confidence level ranging from 80% to 99.5% for Oncosmart3 RSDs.

Supplementary Table 1 (Table S1): Panel-wide statistics for 30 BGs, 3 HWTs and 2 tumor samples.

Supplementary Table 2-1 (Table S2-1): Sensitivity under different confidential levels at AF level from 0.1% to 5% in Oncosmart3 RSDs.

Supplementary Table 2-2 (Table S2-2): Annotations for false positive sites in Oncosmart3 RSDs.

Supplementary Table 2-3 (Table S2-3): Pie chart of occurrence for false positives in Oncosmart3 RSDs.

Supplementary Table 2-4 (Table S2-4): Substitution frequency of false positive sites in Oncosmart3 RSDs.

Supplementary Table 3-1 (Table S3-1): Median of AIC, BIC, r from quantile-quantile plot (QQ plot) and SEE among 10 random samples.

Supplementary Table 3-2 (Table S3-2): Percentages of best fitted distribution among randomly selected 1000 genomic sites for three times in 104 Oncosmart1 patient cfDNA samples..

Supplementary Table 3-3 (Table S3-3): Parameters of best-fitted distribution and AF cutoff values for 265 polishing sites.

Supplementary Table 3-4 (Table S3-4): Percentages of r value above provided r value among 87 polishing sites fitted both by iDES and Our distributions.

Supplementary Table 4-1 (Table S4-1): Detailed performance statistics for VBCALAVD, 5 benchmarked panel-wide callers and reported performances for iDES and ERASE-Seq.

Supplementary Table 4-2 (Table S4-2): Panel wide error-free position percentages of 25 Oncosmart2 BGs and reported percentages of BGs in iDES.

Supplementary Table 5 (Table S5): Annotations for left sites in controls.

Supplementary Table 6 (Table S6): Distribution of controls’ left sites with AF values in multiplexing samples at same sequencing line.

**References**

1. Li H, Durbin R. Fast and accurate long-read alignment with Burrows–Wheeler transform. Bioinformatics. 2010;26(5):589-95.

2. Li H, Handsaker B, Wysoker A, Fennell T, Ruan J, Homer N, et al. The sequence alignment/map format and SAMtools. Bioinformatics. 2009;25(16):2078-9.

3. Lai Z, Markovets A, Ahdesmaki M, Chapman B, Hofmann O, McEwen R, et al. VarDict: a novel and versatile variant caller for next-generation sequencing in cancer research. Nucleic Acids Res. 2016 Jun 20;44(11):e108. PubMed PMID: 27060149. Pubmed Central PMCID: PMC4914105.

4. Newman AM, Lovejoy AF, Klass DM, Kurtz DM, Chabon JJ, Scherer F, et al. Integrated digital error suppression for improved detection of circulating tumor DNA. Nature biotechnology. 2016;34(5):547.

5. Deng Q, Xie B, Wu L, Ji X, Li C, Feng L, et al. Competitive evolution of NSCLC tumor clones and the drug resistance mechanism of first-generation EGFR-TKIs in Chinese NSCLC patients. Heliyon. 2018;4(12):e01031.

6. Kamps-Hughes N, McUsic A, Kurihara L, Harkins TT, Pal P, Ray C, et al. ERASE-Seq: Leveraging replicate measurements to enhance ultralow frequency variant detection in NGS data. PLOS ONE. 2018;13(4):e0195272.

7. McKenna A, Hanna M, Banks E, Sivachenko A, Cibulskis K, Kernytsky A, et al. The Genome Analysis Toolkit: a MapReduce framework for analyzing next-generation DNA sequencing data. Genome Res. 2010 Sep;20(9):1297-303. PubMed PMID: 20644199. Pubmed Central PMCID: PMC2928508.

8. Koboldt DC, Zhang Q, Larson DE, Shen D, McLellan MD, Lin L, et al. VarScan 2: somatic mutation and copy number alteration discovery in cancer by exome sequencing. Genome Res. 2012 Mar;22(3):568-76. PubMed PMID: 22300766. Pubmed Central PMCID: PMC3290792.

9. Cibulskis K, Lawrence MS, Carter SL, Sivachenko A, Jaffe D, Sougnez C, et al. Sensitive detection of somatic point mutations in impure and heterogeneous cancer samples. Nat Biotechnol. 2013 Mar;31(3):213-9. PubMed PMID: 23396013. Pubmed Central PMCID: PMC3833702.

10. Kockan C, Hach F, Sarrafi I, Bell RH, McConeghy B, Beja K, et al. SiNVICT: ultra-sensitive detection of single nucleotide variants and indels in circulating tumour DNA. Bioinformatics. 2017 Jan 1;33(1):26-34. PubMed PMID: 27531099.

11. Dohm JC, Lottaz C, Borodina T, Himmelbauer H. Substantial biases in ultra-short read data sets from high-throughput DNA sequencing. Nucleic acids research. 2008;36(16):e105.

12. Fuller CW, Middendorf LR, Benner SA, Church GM, Harris T, Huang X, et al. The challenges of sequencing by synthesis. Nature biotechnology. 2009;27(11):1013.
